# Single‐Molecule Characterization of Bacterial Factor‐Dependent Transcription Activation by Rob

**DOI:** 10.1002/advs.76334

**Published:** 2026-07-06

**Authors:** Yuqiong Zhang, Weijing Han, Jing Shi, Lisha Wang, Cuijuan Teng, Wei Lin, Jun Fan, Shuang Wang

**Affiliations:** ^1^ Institute of Fundamental and Frontier Sciences University of Electronic Science and Technology of China Chengdu Sichuan China; ^2^ Chinese Medicine Guangdong Laboratory Guangzhou University of Chinese Medicine Hengqin Guangdong China; ^3^ School of Medicine Department of Infectious Diseases Nanjing University of Chinese Medicine Nanjing Drum Tower Hospital Nanjing Jiangsu China; ^4^ Shandong Huayu University of Technology Dezhou Shandong China

**Keywords:** bacterial transcription, biology, rna polymerase, single‐molecule experiment, single‐molecule fret, small molecule ligand, transcription activation, transcription factor, transcription initiation

## Abstract

Factor‐dependent transcription activation is a key process of transcription regulation, involving complex interactions between multiple regulatory factors, RNA polymerase (RNAP), and promoter DNA, whose mechanism remains unclear. Here, we use single‐molecule magnetic trapping assays and fluorescence assays to characterize the kinetics of *Escherichia coli* (*E. coli*) RNAP transcription under the regulation of a pleiotropic AraC/XylS family factor, Rob, and elucidate their underlying mechanisms. We find that Rob prebinds RNAP holoenzyme to form a binary complex to facilitate promoter search. Upon promoter recognition, Rob enhances RNAP‐mediated promoter DNA unwinding to facilitate promoter escape and transition into transcription elongation. Rob activity is diversely regulated by small ligands, implying important roles of Rob C‐terminal domain (CTD) on its regulatory function. Our findings shed light on the mechanisms of Rob‐dependent transcription activation, which may provide insights into other transcription regulators in prokaryotes as well as eukaryotes.

## Graphical abstract

Factor‐dependent transcription activation is crucial and involves complex interactions between transcription factors, RNA polymerase, and promoter DNA. Using single‐molecule assays, we show that Rob prebinds RNAP to facilitate promoter search and enhances DNA unwinding and promoter escape. Ligand regulation of Rob activity reveals diverse modes of transcriptional control.

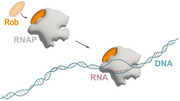

## 1 Introduction

Factor‐dependent transcription activation is one of the most important pathways that bacteria use to regulate gene expression in response to environmental change. Bacterial transcription is mainly accomplished by an RNA polymerase (RNAP) under the regulation of various transcription factors, where RNAP‐mediated promoter recognition and initial transcription are likely the key steps [1]. The RNAP core enzyme consists of five subunits (α_II_ββ′ω) and associates with a *σ* factor to form a holoenzyme, enabling promoter recognition and transcription initiation [2, 3]. The RNAP holoenzyme specifically binds to promoter DNA through interactions between the α C‐terminal domain (αCTD) and the upstream element, ‐35 element and the*σ* ^70^ region 4 (*σ* ^70^R4), and ‐10 element and the *σ* ^70^ region 2 (*σ* ^70^R2) [4, 5], leading to downstream DNA unwinding and RNAP conformational changes to form open promoter complex (RPo) [5, 6, 7, 8, 9] and subsequently the initial transcription complex (RPitc) [10, 11, 12].

Various transcription factors interact with RNAP to regulate bacterial transcription levels in response to environmental stresses [1, 13], among which Rob is a member of the AraC/XylS family regulators responsible for oxidative and multidrug stresses [14, 15]. Rob comprises an N‐terminal domain (NTD) and a distinctive C‐terminal domain (CTD) [16]. Rob NTD shares structural similarities with other AraC/XylS family members, SoxS, MarA, and TetD [17], and has been found to recognize and bind to the Rob binding box (Rob‐box) of Class II promoters, where the Rob binding box partially overlaps with the ‐35 element of the promoter [16]. In addition, Rob NTD binds to the *σ* ^70^R4 of RNAP holoenzyme, and the Rob binding box overlaps with the ‐35 element to occlude the interaction between the *σ* ^70^R4 and the ‐35 element of the promoter, as observed in the RPo complex, similarly to its homolog SoxS [7, 18, 19]. Due to these interactions, a “prerecruitment” mechanism has been proposed for the promoter search step of SoxS [20]. However, whether a similar mechanism is common to Rob or other AraC/XylS family members remains an open question. In consistent with MarA, Rob bends promoter DNA through these interactions, with the aid of the accessory acidic loop of Rob CTD [19, 21, 22]. As a distinctive domain from other AraC/XylS family members, Rob CTD possesses a Gyrl‐like architecture composed of α helices and β sheets forming a ligand binding pocket, which may be capable of binding to small ligands [19, 23]. The Rob CTD can prevent degradation in vivo, possibly by enclosing the NTD by the CTD and allowing the NTD to be accessible for DNA binding upon ligand binding to Rob CTD, indicating a “sequestration‐dispersal” mechanism for Rob regulation [24]. However, the biological insights of the DNA‐bending property and the CTD functions of Rob need to be addressed.

Single‐molecule assays have been widely applied to the studies of prokaryotic transcription and regulation [25, 26, 27, 28, 29]. Here, we take the advantages of the single‐molecule magnetic trapping assays and single‐molecule fluorescence assays to characterize the kinetic rate at which RNAP molecules search for promoter and the efficiency of the RPo formation under the effect of the AraC/XylS family regulator Rob, based on which a promoter search mechanism of Rob is proposed. In addition, we characterize the kinetic rate, the apparent DNA unwinding, and DNA bending during RNAP initial transcription under Rob regulation to infer the mechanisms of Rob DNA bending on initial transcription. We further determine the effect of three small ligands, quinoline carboxamide, chenodeoxycholic acid, and 2,2′‐bipyridine, on different steps of Rob‐dependent transcription activation, to elucidate multiple effects of Rob CTD and the small ligands on transcription. Our results provide a kinetic and quantitative view of Rob‐dependent transcription activation and may provide insight into the mechanisms of factor‐dependent transcription activation in prokaryotes and eukaryotes.

## 2 Results

### 2.1 Rob Facilitates Promoter Search by *E. coli* RNAP

**FIGURE 1:**
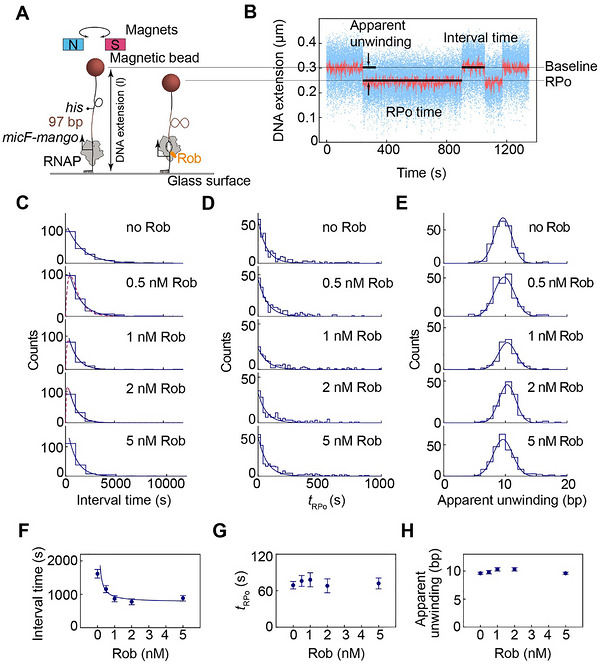
Rob‐dependent formation of RNAP‐promoter open complex characterized via single‐molecule magnetic trapping assays. (A) Schematic of the single‐molecule magnetic trapping assays. A 2.2‐kb linear DNA, containing a *micF‐mango* promoter followed by a 97 bp transcription unit and a *his* terminator, is tethered between a magnetic bead and a glass surface. The DNA molecule is slightly extended and positively supercoiled, thus allowing the measurements of promoter unwinding by an RNAP to form an RNAP‐promoter open complex (RPo). (B) A typical trajectory representing RPo formation reflected by a discrete DNA extension decrease below the baseline for about 50 nm, i.e., DNA unwinding for one helix turn. Lifetimes of RPo states as well as their intervals are analyzed. The apparent promoter unwinding of each RPo state is analyzed as described in Methods. (C, D) Duration histograms of Interval time (C) and *t*_RPo_ (D) are each fit to a single exponential function, yielding time constants of 1615 ± 128 s, 1155 ± 106 s, 866 ± 92 s, 773 ± 87 s and 882 ± 89 s (SEM, *N* = 200, 173, 121, 129 and 171 for C, navy lines), and 68.6 ± 6.2 s, 75.6 ± 8.7 s, 77.5 ± 11.5 s, 68.1 ± 11.5 s, and 72.2 ± 9.0 s (SEM, *N* = 255, 226, 137, 164 and 248 for D, navy lines) for 0 nm, 0.5 nm, 1 nm, 2 nm, and 5 nm Rob, respectively. The data in (C) were then fit to the single‐molecule Michaelis‐Menten model (equation 3), yielding *k*_1_ = 6.7 ± 0.2 M^−12^s^−1^ and *k*_2_ = 0.0012 ± 0.0001 s^−1^ for 0.5 nm, 1 nm, and 2 nm Rob, respectively (dashed pink line). (E) Histograms of apparent unwinding of each transcription bubble at RPo state are fit to a single‐Gaussian function giving peaks at 9.6 ± 0.2 bp, 9.8 ± 0.3 bp, 10.3 ± 0.3 bp, 10.3 ± 0.3 bp, and 9.6 ± 0.2 bp (SEM, *N* = 255, 226, 137, 164 and 248) for 0 nm, 0.5 nm, 1 nm, 2 nm, and 5 nm Rob, respectively. The fit results of (C–E) are summarized in (F–H), respectively. The data in (F) is fit to the Michaelis‐Menten model (Equation 2), yielding *t*_0_ = 766 ± 72 s and *K*_m_ = 0.22 ± 0.11 nm (SEM).

To interpret the mechanisms of factor‐dependent transcription activation, we take the *E. coli* pleiotropic AraC/XylS family factor Rob and characterize its regulatory effect on the kinetics of RNAP transcription via single‐molecule magnetic trapping assays. A 2.2 kb DNA construct, centrally containing the Rob binding box and a *micF‐mango* promoter, followed by a 97 bp transcription unit and an *E. coli his* terminator, is tethered between a magnetic bead and a glass surface (Figure 1A). DNA molecules are extended at *F* = 0.3 pN (1 pN = 10^−12^ N) and positively supercoiled, allowing real‐time characterization of transcription initiation, elongation, and termination reflected by the mechanical responses of DNA imposed by RNAP molecules [10, 25, 30].

Addition of 2 nm RNAP led to the formation of RNAP‐promoter open complex (RPo), reflected by the abrupt DNA extension decrease for about 50 nm below the baseline, lasting for a duration prior to returning to the baseline [30] (Figure 1B). To quantitatively describe the kinetics of RPo formation, we analyzed the lifetime of each RPo state and the time for RPo formation, which is the interval time between adjacent RPo states, and fit these results to a single exponential function yielding the mean time constant of *t*_RPo_ = 68.0 ± 12.4 s and interval time of 1615 ± 285 s (standard error of the mean, SEM, Figure 1C.D), respectively. To characterize the effect of Rob on the kinetics of RPo formation, we titrated Rob concentration from 0.5 nm to 5 nm against 2 nm RNAPs and analyzed the dependency of the interval time on Rob concentration. A gradually reduced time constant for RPo formation was observed as Rob concentration increases (Figure 1C,F and Table 1). From Figure 1F, the interval time can be separated into a Rob concentration‐dependent process and a Rob concentration‐independent process. We use the single‐molecule Michaelis‐Menten model to fit the data and obtained *k*_1_ = 6.7 ± 0.2 M^−12^s^−1^ for the [Rob] dependent step and *k*_2_ = 0.0012 ± 0.0001 s^−1^ for the [Rob] independent step. The mean interval time reaches a minimum value at 2 nm Rob (against 2 nm holo RNAPs) and cannot further decrease as Rob concentration increases (Figure 1F), consistent with a single Rob action on RPo formation as previously reported [19]. This result can be described by the Michaelis‐Menten model with a minimum time of *t*_0_ = 766 ± 72 s and *K*_m_ = 0.22 ± 0.11 nm. By constraining *k*_2_ = 1/*t*_0_, we obtained consistent *k*_1_ of 5.5 ± 1.0 M^−12^s^−1^ (Figure S1). These results suggest a two‐step model for Rob promotion on RNAP searching promoters. The mean *t*_RPo_ values are statistically identical at different Rob concentrations, suggesting that the dissociation kinetics of the RPo state are possibly not regulated by Rob (Figure 1D,G). The transcription bubble size of each RPo state at each Rob concentration was analyzed by averaging the apparent DNA unwinding over the total RPo time (Figure 1B). Fitting these histograms to a single‐Gaussian function yields statistically identical values (Figure 1E,H), suggesting two possibilities: either Rob doesn't regulate the transcription bubble size of the RPo state, or Rob dissociates as RPo formation, which can be excluded because of the observation of Rob in the Rob‐RNAP‐DNA structure [19].

**TABLE 1:** Summary of the fit results for interval time, *t*_RPo_, and transcription bubble size at different Rob concentrations.

	no Rob	0.5 nm Rob	1 nm Rob	2 nm Rob	5 nm Rob
Interval time	1615 ± 128 s	1155 ± 106 s	866 ± 92 s	773 ± 87s	882 ± 89 s
*t*_RPo_	68.6 ± 6.2 s	75.6 ± 8.7 s	77.5 ± 11.5 s	68.1 ± 11.5 s	72.2 ± 9.0 s
Apparent unwinding	9.6 ± 0.2 bp	9.8 ± 0.3 bp	10.3 ± 0.3 bp	10.3 ± 0.3 bp	9.6 ± 0.2 bp

Overall, our results suggest that, although it barely affects the transcription bubble size and the stability of the RPo state, a single Rob acts to facilitate promoter search by *E. coli* RNAP. Because Rob can bind to both the conserved Rob binding box near the ‐35 element of the class II promoter and the αCTD of RNAP in the Rob‐RNAP‐DNA activation complex [19], it is possible that Rob may facilitate promoter search either by prebinding to RNAP to form a binary complex or by prebinding to the Rob binding box of DNA to recruit RNAP holoenzyme. Determination of either of these two possibilities may provide deeper interpretations of the mechanisms of factor‐dependent transcription activation, which may be common to other transcription regulators.

### 2.2 Rob Prebinds RNAP as a Binary Complex to Facilitate Promoter Search

**FIGURE 2:**
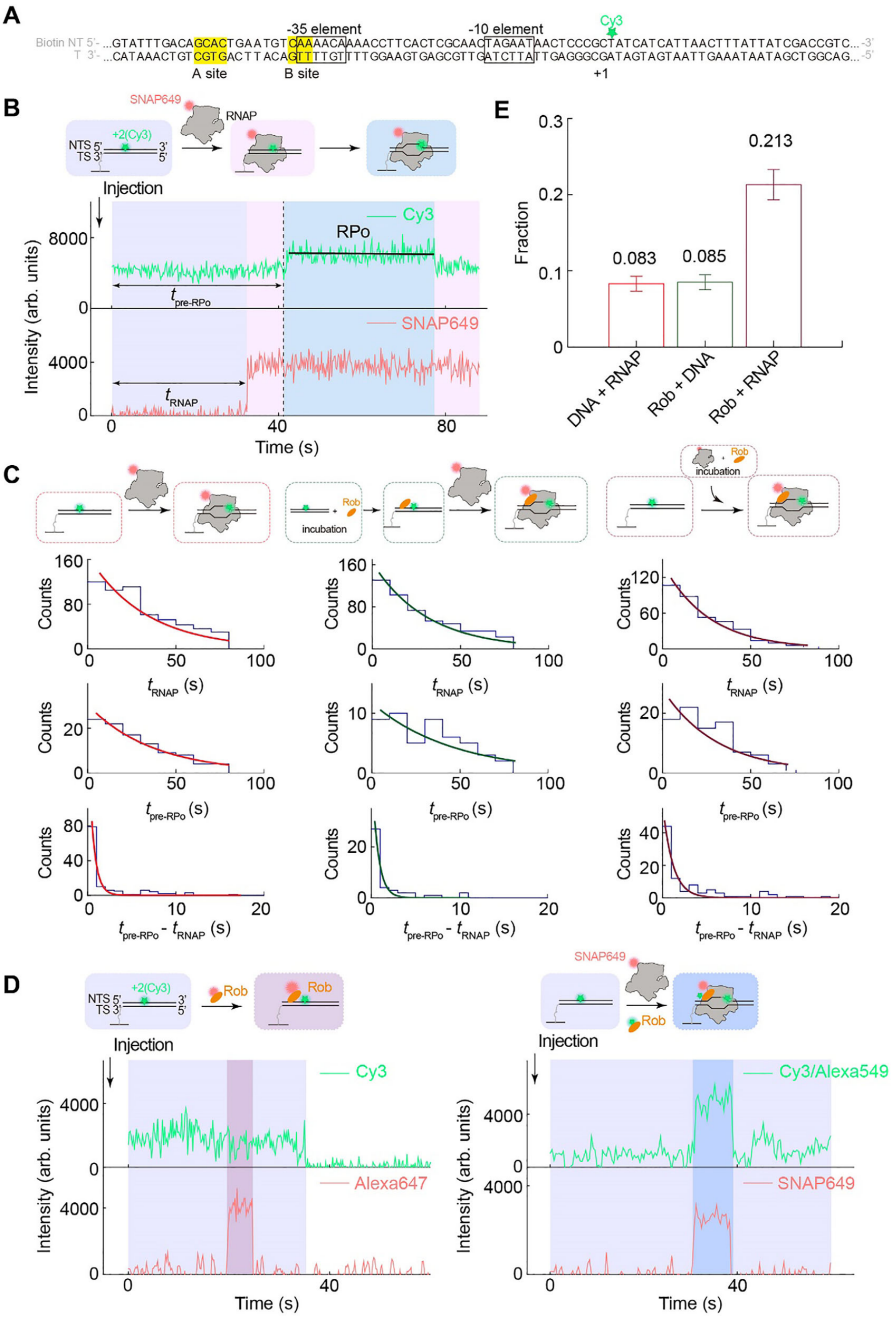
Characterization of the Rob binding kinetics via single‐molecule fluorescence assays. (A) DNA sequence representing the −35 and −10 elements of *micF‐mango* promoter, the A and B sites for Rob binding, and Cy3 labeling at +2 position of the nontemplate strand for single‐molecule fluorescence assays. (B) Schematic of the assays where DNA‐Cy3 was tethered to a PEG surface for characterization of RNAP‐SNAP649 binding and RPo formation (upper panel). Typical trajectories showing RNAP binding as reflected by the appearance of RNAP‐SNAP649, and the RPo formation as reflected by Cy3 PIFE. (C) Schematic of experimental design: addition of RNAP‐SNAP649 to surface‐tethered DNA‐Cy3 (left), RNAP‐SNAP649 is introduced after DNA‐Cy3 preincubated with 2 nm Rob (middle), RNAP‐SNAP649 is preincubated with 2 nm Rob and then added to surface‐tethered DNA‐Cy3 (right). Histograms of the time for RNAP‐SNAP649 appearance (*t*_RNAP_), RPo formation (*t*_pre‐RPo_), and the subtraction between these two times (*t*_pre‐RPo_—*t*_RNAP_) are plotted for three experiments. Fitting each histogram to a single exponential function gives mean RPo formation time of *t*_pre‐RPo_ = 37.2 ± 5.9 s, 45.8 ± 12.1 s, and 30.8 ± 4.7 s (SEM, *N* = 104, 50, and 89), respectively; mean RNAP appearance time of *t*_RNAP_ = 33.2 ± 2.0 s, 31.1 ± 1.9 s, and 25.6 ± 1.7 s (SEM, *N* = 562, 503, and 361), respectively; and the differences between *t*_pre‐RPo_ and *t*_RNAP_ of 0.6 ± 0.1 s, 0.7 ± 0.1 s, and 1.1 ± 0.2 s (SEM, *N* = 123, 44, and 94), respectively. (D) Schematic of the assays to characterize the formation of the Rob‐DNA complex (left panel) and the Rob‐RNAP heterodimer (right panel). Typical trajectories represent the surface‐tethered DNA‐Cy3 bound with Rob‐Alexa647 (left panel), and the surface‐tethered DNA‐Cy3 bound with Rob‐Alexa546/RNAP‐SNAP649 complex (right panel). (E) The fraction is calculated as the number of RPo formation events over the number of RNAP‐SNAP649 binding events for three experiments: 123 out of 1480, 50 out of 584, and 89 out of 418 events, respectively.

To investigate how Rob facilitates promoter search, we use short DNA tethers containing the essential elements of Rob binding box and a *micF‐mango* promoter to exclude the possibility of nonspecific binding of either of these two proteins to the long DNA tethers in single‐molecule magnetic trapping assays, and employ single‐molecule fluorescence assays to characterize the kinetics of fluorescently labeled RNAP binding to promoter DNA and RPo formation under the effect of Rob. Fluorescently labeled DNA (Cy3 at +2 position of the non‐template strand, DNA‐Cy3), containing a Rob binding box (A, B sites) and a *micF‐mango* promoter, was tethered to a PEG surface (Figure 2A,B). Upon addition of SNAP649‐labeled RNAP (RNAP‐SNAP649), alternative illumination of a 532 nm and a 640 nm laser is applied. The Cy3 and SNAP649 fluorescence were collected on an EMCCD camera, and the time for RNAP binding to DNA (*t*_RNAP_) was reflected by the appearance of RNAP‐SNAP649 fluorescence (bottom panel in Figure 2B), and the time for RPo formation (*t*_pre‐RPo_) was reflected by the protein‐induced fluorescence enhancement (PIFE) [31, 32] of Cy3 fluorophore were monitored in real time (middle panel in Figure 2B). Single‐molecule fluorescence experiments were designed so that Rob was preincubated separately with either RNAP‐SNAP649 or surface‐tethered DNA‐Cy3, followed by introduction to surface‐tethered DNA‐Cy3 or RNAP‐SNAP649, respectively. Each condition aims to mimic Rob prebinding to RNAP or DNA during promoter search, respectively. The kinetics achieved from these two experiments were then compared to those from the control experiment of direct introduction of RNAP‐SNAP649 to surface‐tethered DNA‐Cy3 in the absence of Rob (upper panels in Figure 2C).

In the control experiment, we measured the time for RNAP binding to DNA and the time before RPo formation, respectively. Fitting these data to a single exponential function yields a mean time of *t*_pre‐RPo_ = 37.2 ± 5.9 s (SEM) before RPo formation and *t*_RNAP_ = 33.2 ± 2.0 s (SEM) for RNAP binding, respectively (left panels in Figure 2C and Table 2). The difference between each pair of these two times was calculated and then fit to a single exponential function, yielding a mean time of *t*_pre‐RPo_—*t*_RNAP_ = 0.6 ± 0.1 s (SEM). When preincubating Rob with DNA‐Cy3 followed by being tethered to a PEG surface and addition of RNAP‐SNAP649, i.e., mimicking Rob prebinding to promoter DNA to recruit RNAP, the mean times for RPo formation, RNAP binding, and their differences are statistically identical to those values of the control experiment (middle panels in Figure 2C). Rob binding to DNA‐Cy3 can be directly observed (left panel in Figure 2D; Figure S2). These results suggest that Rob may bind to promoter DNA but cannot accelerate the kinetics for RNAP recruitment as previously discussed [33]. When preincubating Rob with RNAP followed by addition to surface‐tethered DNA‐Cy3, i.e., mimicking Rob prebinding to RNAP to search for a promoter, the mean time for RPo formation is slightly reduced compared to those values of the above experiments (right panels in Figure 2C). More obviously, the mean time for RNAP binding to promoter DNA is significantly reduced, suggesting that preincubation of Rob with RNAP may allow them to form a Rob‐RNAP binary complex, which facilitates the kinetics of promoter search by RNAP. Consistently, Rob and RNAP can form a complex as observed via single‐molecule fluorescence assays (Figure 2D; Figure S2). The mean time for *t*_pre‐RPo_—*t*_RNAP_ is slightly enhanced, which may suggest a different kinetics of RPo formation by Rob‐RNAP binary complex. The fraction of RPo formation calculated as the number of RPo formation events over the number of RNAP binding events is significantly higher when preincubating Rob with RNAP than the values from the other experiments, which, again, supports that Rob prebinding to RNAP can facilitate RPo formation (Figure 2E).

**TABLE 2:** Summary of the fit results for RPo formation (*t*_pre‐RPo_), RNAP‐SNAP649 appearance (*t*_RNAP_), and the subtraction between these two times (*t*_pre‐RPo_—*t*_RNAP_) under different incubation conditions.

	DNA + RNAP	Incubation Rob + DNA	Incubation Rob + RNAP
*t*_pre‐RPo_	37.2 ± 5.9 s	45.8 ± 12.1 s	30.8 ± 4.7 s
*t*_RNAP_	33.2 ± 2.0 s	31.1 ± 1.9 s	25.6 ± 1.7 s
*t*_pre‐RPo_—*t*_RNAP_	0.6 ± 0.1 s	0.7 ± 0.1 s	1.1 ± 0.2 s

Single‐molecule fluorescence results achieved in this section are consistent with the single‐molecule magnetic trapping results from the previous section. Taking these results together, we propose a model that Rob prebinds RNAP to form a binary complex, i.e., Rob‐RNAP complex, to facilitate promoter search and thus activate transcription. This is possibly because the Rob‐RNAP complex possesses a higher binding affinity to promoter DNA than a single RNAP due to the interactions between the Rob‐RNAP complex and the Rob‐binding box/*micF‐mango* promoter.

### 2.3 Rob Facilitates RNAP Entry into Initial Transcription

Previous structural reports suggest that Rob remains bound with RNAP and promoter rather than getting dissociated after RPo formation [19]. It would therefore be interesting to determine whether bound Rob affects the kinetics of subsequent steps of transcription initiation, a highly regulated step that RNAP unwinds the downstream promoter DNA and uses it as a template for RNA synthesis, forming the initial transcription complex (RPitc) [10, 11]. We then used single‐molecule fluorescence assays [28] to characterize the kinetics of RPitc formation, as well as the associated conformational changes under the effect of Rob.

**FIGURE 3:**
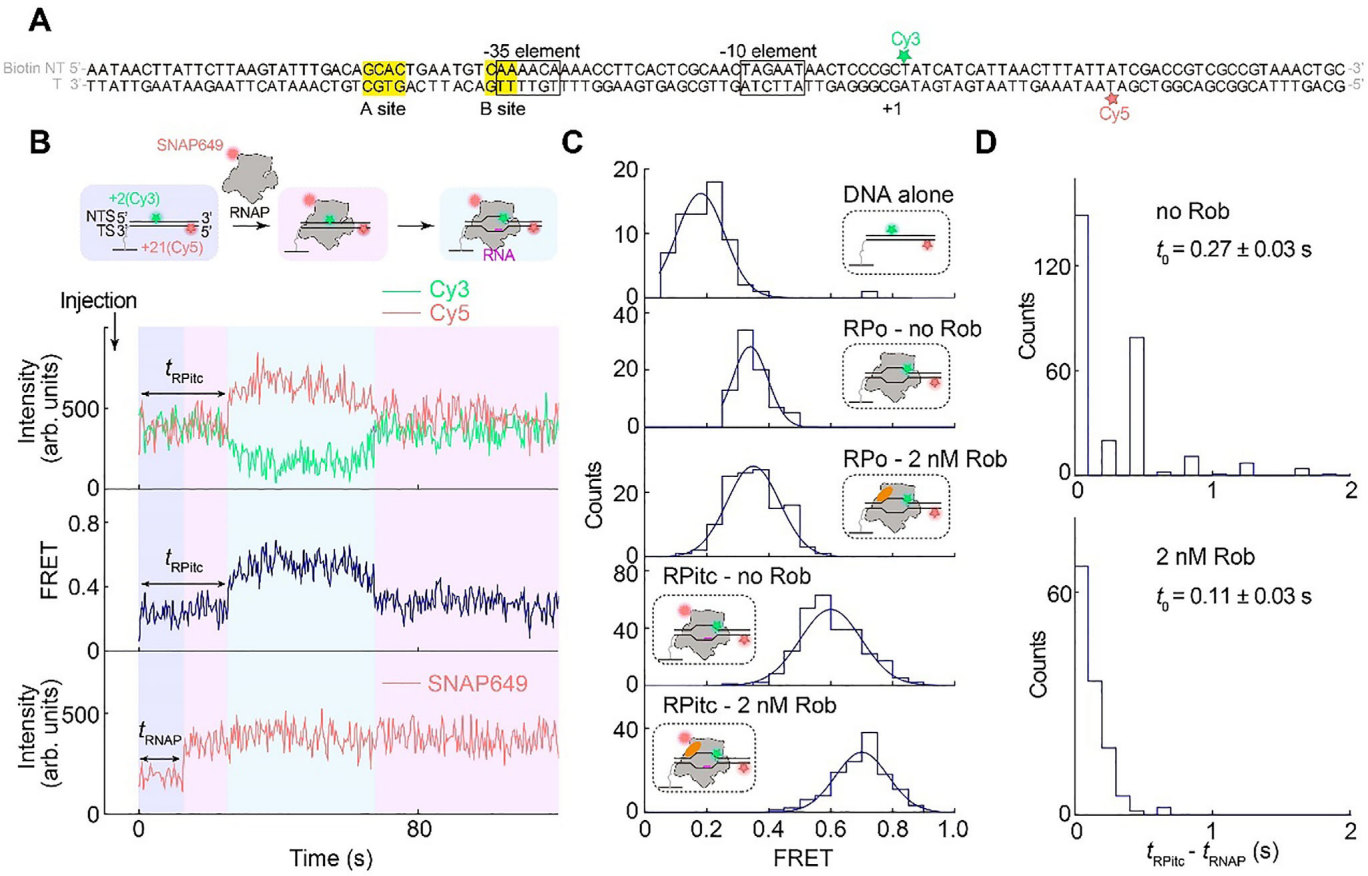
Characterization of Rob effect on RPitc formation via single‐molecule fluorescence assays. (A) The same DNA is used as in Figure 2A, except for an additional Cy5 labeling at the +21 position of the template strand. (B) Schematic for single‐molecule fluorescence assays, where DNA molecules were tethered to a PEG surface for the measurements of the Rob effect on initial transcription upon addition of RNAP‐SNAP649 (upper panel). In the lower panels, typical trajectories representing RPitc formation as reflected by the FRET increase above ∼0.4 (navy in the bottom second panel) after RNAP appearance (SNAP649 fluorescence in the bottom panel). (C) Mean FRET value of each trajectory was analyzed in the conditions of DNA alone, RPo without Rob, RPo with 2 nm Rob, RPitc without Rob, and RPitc with 2 nm Rob from top to bottom panels, respectively. Fitting each histogram to a single Gaussian function gives peaks at 0.18 ± 0.03, 0.34 ± 0.02, 0.35 ± 0.01, 0.6 ± 0.01, and 0.7 ± 0.02 (SEM, *N* = 65, 80, 127, 277, and 130 events). (D) Histograms of the time differences between the appearance of RNAP and RPitc are shown with a mean time constant of 0.27 ± 0.03 s (SEM, *N* = 277) in the absence of Rob (upper panel) and 0.11 ± 0.03 s (SEM, *N* = 130) in the presence of 2 nm Rob (lower panel).

Fluorescently labeled DNA‐Cy3/Cy5 (Cy3 at +2 position of non‐template strand and Cy5 at +21 position of template strand) is tethered to a PEG surface (Figure 3A,B). Cy3 and Cy5 fluorophores are illuminated alternatively, and their fluorescence intensities are imaged separately on an EMCCD camera. DNA alone gives a mean FRET value of 0.18 ± 0.03 (SEM) representing a Cy3/Cy5 distance separated by 19 bp DNA duplex (top panel in Figure 3C; Figure S3). Addition of 2 nm RNAP caused the mean FRET value to increase to 0.34 ± 0.02 (SEM), reflecting a reduction in Cy3/Cy5 distance due to promoter DNA unwinding during RPo formation [28] (top second panel in Figure 3C; Figure S4). Extra presence of 2 nm Rob doesn't change this FRET value (0.35 ± 0.01, SEM), suggesting no detectable effect of Rob on promoter DNA unwinding or rewinding (top third panel in Figure 3C; Figure S5). Addition of 2 nM RNAP and 100 µM NTPs (100 µM each of UTP, ATP, GTP, and CTP, respectively) led to RPitc formation, reflected by a significant FRET increase higher than 0.34, i.e., the FRET value of the RPo state (bottom second panels in Figure 3B,C; Figure S6). The FRET values are averaged over each RPitc state separately, and the histogram is fit to a single‐Gaussian function, yielding a peak at 0.6 ± 0.01 (SEM). This FRET value is higher than that of the RPo state (0.34), confirming RPitc formation. An extra addition of 2 nm Rob increased the mean FRET value to 0.70 ± 0.02 (SEM, bottom panel in Figure 3C; Figure S7). Because Cy3 and Cy5 are labeled downstream of the −10 element of the promoter, this result suggests a further promoter DNA unwinding but not DNA bending caused by Rob during RPitc state. How Rob modulates DNA unwinding of the RPitc state will be further characterized by using single‐molecule magnetic trapping assays and discussed in the next section. The time difference between RPitc formation and RNAP binding to promoter DNA was observed (bottom panel in Figure 3B) and plotted in histograms in the absence and presence of 2 nm Rob, respectively (Figure 3D). The mean time constant is obtained by averaging each of the lifetime events, yielding a slightly reduced time constant under the effect of 2 nm Rob (Figure 3D). These results suggest that Rob may slightly facilitate entry into transcription initiation after RNAP binds to promoter DNA.

### 2.4 Rob Enhances DNA Unwinding to Facilitate RNAP Escaping from the Promoter

Following transcription initiation, RNAP often undergoes multiple cycles of abortive RNA synthesis, producing and releasing short transcripts. Until generating a sufficiently long RNA transcript, RNAP escapes from the promoter and enters the elongation state, i.e., productive initiation [12, 34, 35]. The kinetics of successful promoter escape of RNAP, especially under the regulation of transcription factors, modulate RNA generation of downstream genes. To gain further insight into whether and how Rob regulates RNAP escaping from the promoter, we characterized the kinetic rate and transcription bubble sizes of RNAP productive transcription initiation in the absence and presence of Rob via single‐molecule magnetic trapping assays due to its ease to distinguish productive and abortive transcription initiation events [25, 28].

**FIGURE 4:**
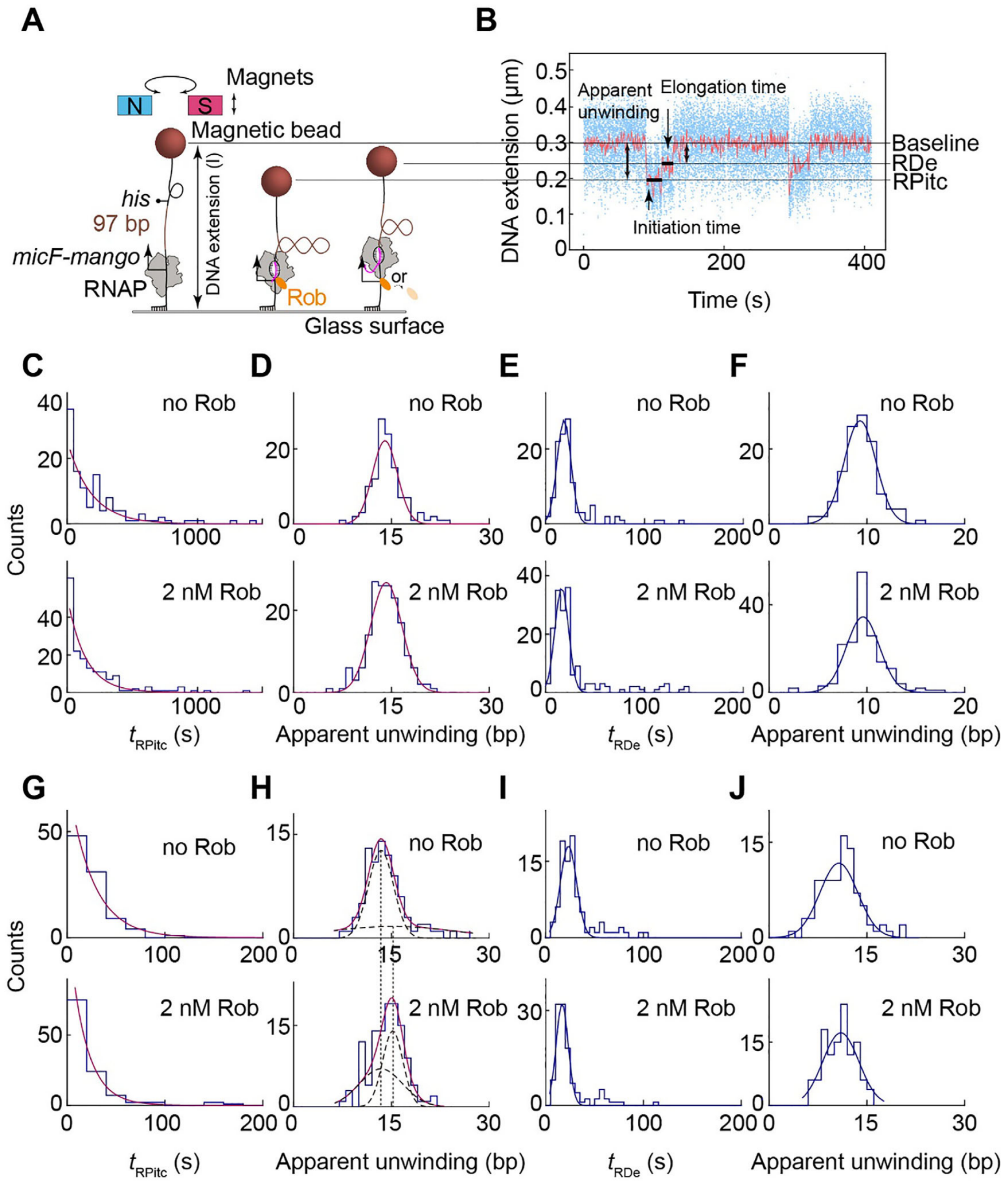
Characterization of the Rob effect on transcription initiation and elongation under positively and negatively supercoiled DNA via single‐molecule magnetic trapping assays. (A) The presence of NTP allows RNAP to unwind the DNA sequence downstream of the promoter to form the RNAP‐promoter initial transcription complex (RPitc), the RNAP‐DNA elongation complex (RDe), and then termination. (B) A typical trajectory representing the sequential formation of RPitc and RDe states under positively supercoiled DNA, and data are presented in (C–F). (C) Histograms of the lifetimes of each RPitc state are fit to a single exponential function, giving time constants of 183 ± 24 s and 140 ± 13 s (SEM, *N* = 119, 171) for 0 and 2 nm Rob, respectively. (D) Histograms of the transcription bubble sizes are fit to a single‐Gaussian function, yielding peaks at 14.0 ± 0.4 bp and 14.2 ± 0.4 bp (SEM, *N* = 119, 171) for 0 and 2 nm Rob, respectively. (E) Histograms of the lifetimes of each RDe state are fit to a single‐Gaussian function, giving peaks at 18.6 ± 1.5 s and 16.2 ± 1.3 s (SEM, *N* = 119, 171) for 0 nm and 2 nm Rob, respectively. (F) Histograms of the transcription bubble sizes are fit to a single‐Gaussian function, yielding peaks at 9.3 ± 0.4 bp and 9.6 ± 0.3 bp (SEM, *N* = 119, 171) for 0 and 2 nm Rob, respectively. Data achieved from negative supercoiled DNA is represented in (G–J). (G) Histograms of the lifetime of each RPitc state are fit to a single exponential function, giving time constants of 25.2 ± 3.2 s and 17.4 ± 2.0 s (SEM, *N* = 94, 118) for 0 and 2 nm Rob, respectively. (H) Histograms of the transcription bubble sizes are distributed and globally fit to a double‐Gaussian function, yielding two peaks at 13.4 ± 0.3 bp and 15.2 ± 0.3 bp for 0 nm and 2 nm Rob, respectively, with amplitudes of 12.7 ± 2.3 and 1.7 ± 1.1 for 0 nm Rob, and 7.0 ± 3.2 and 14.0 ± 3.7 for 2 nm Rob for the two peaks at 13.4 and 15.2, respectively (SEM, *N* = 94, 118). (I) Histograms of the lifetime of each RDe state are fit to a single‐Gaussian function, giving peaks at 23.4 ± 2.1 s and 16.8 ± 1.5 s (SEM, *N* = 94, 118) for 0 and 2 nm Rob, respectively. (J) Histograms of the transcription bubble sizes are fit to a single‐Gaussian function, yielding peaks at 10.7 ± 0.7 bp and 10.9 ± 0.7 bp (SEM, *N* = 94, 118) for 0 and 2 nm Rob, respectively. Additional data for negative supercoiled DNA are represented in Figure S9.

The same 2.2‐kb DNA construct as in the first section is used to generate positively supercoiled DNA and subjected to single‐molecule magnetic trapping assays [10] (Figure 4A). Upon addition of 2 nm RNAP and 100 µm NTPs, we observed abrupt DNA extension decrease for more than 50 nm below the baseline, reflecting RPitc formation, followed by returning to about 50 nm below the baseline, reflecting entry into elongation state, i.e., RNAP‐DNA elongation complex (RDe, Figure 4B). The RDe state returns to baseline when RNAP encounters the *his* terminator. In the absence of Rob, the lifetimes of each RPitc state prior to entering a RDe state were analyzed and fit to a single exponential function, yielding a time constant of 183 ± 24 s (SEM, upper panel in Figure 4C). The presence of 2 nm Rob slightly reduced the time constant of the RPitc state (140 ± 13 s, SEM, lower panel in Figure 4C), suggesting that Rob does regulate the kinetics of RNAP escaping from the promoter. To find out whether this regulation is related to the Rob effect on RNAP‐mediated DNA unwinding or rewinding activity, we characterized transcription bubble sizes of each RPitc state and obtained statistically identical transcription bubble sizes for 0 nm and 2 nm Rob under positively supercoiled DNA. Because of the observation of the different FRET values of the RPitc states between 0 nm and 2 nm Rob in the previous section (Figure 3C), we further performed single‐molecule magnetic trapping assays on negatively supercoiled DNA, observing facilitation of Rob in the kinetics of RPitc state and a slightly enhanced transcription bubble size by around 2 bases (Figure 4G,H; Figure S9). This result supports that Rob may regulate transcription bubble size of linear DNA and negatively supercoiled DNA, but not positively supercoiled DNA. Although no Rob‐RPitc structure is available, our result is consistent with the Rob configuration that bridges RNAP and DNA in the RPo structure [19], where the interactions of Rob‐RNAP and Rob‐DNA may promote DNA unwinding for RNAP to enter transcription elongation state [34].

The elongation time and transcription bubble size at each condition in the absence or presence of 2 nm Rob were analyzed and fit to a single‐Gaussian function separately, yielding statistically identical time constants and transcription bubble sizes for positively supercoiled DNA (Figure 4E,F). Interestingly, the mean elongation time is slightly reduced by Rob, but the transcription bubble size remains statistically identical on negatively supercoiled DNA (Figure 4I,J; Figure S9), suggesting that transcription elongation may be regulated by Rob under negatively supercoiled DNA.

### 2.5 Rob CTD‐Binding Ligands Regulate Rob‐Dependent Transcription Activation

The binding of small ligands to transcription factors enables alternative regulatory mechanisms of transcription in response to environmental stress [24]. Unlike other AraC/XylS regulators, Rob possesses a Gyrl‐like CTD capable of binding small ligands [19, 36, 37]. However, how ligand binding to Rob CTD affects transcription kinetics is unclear.

**FIGURE 5:**
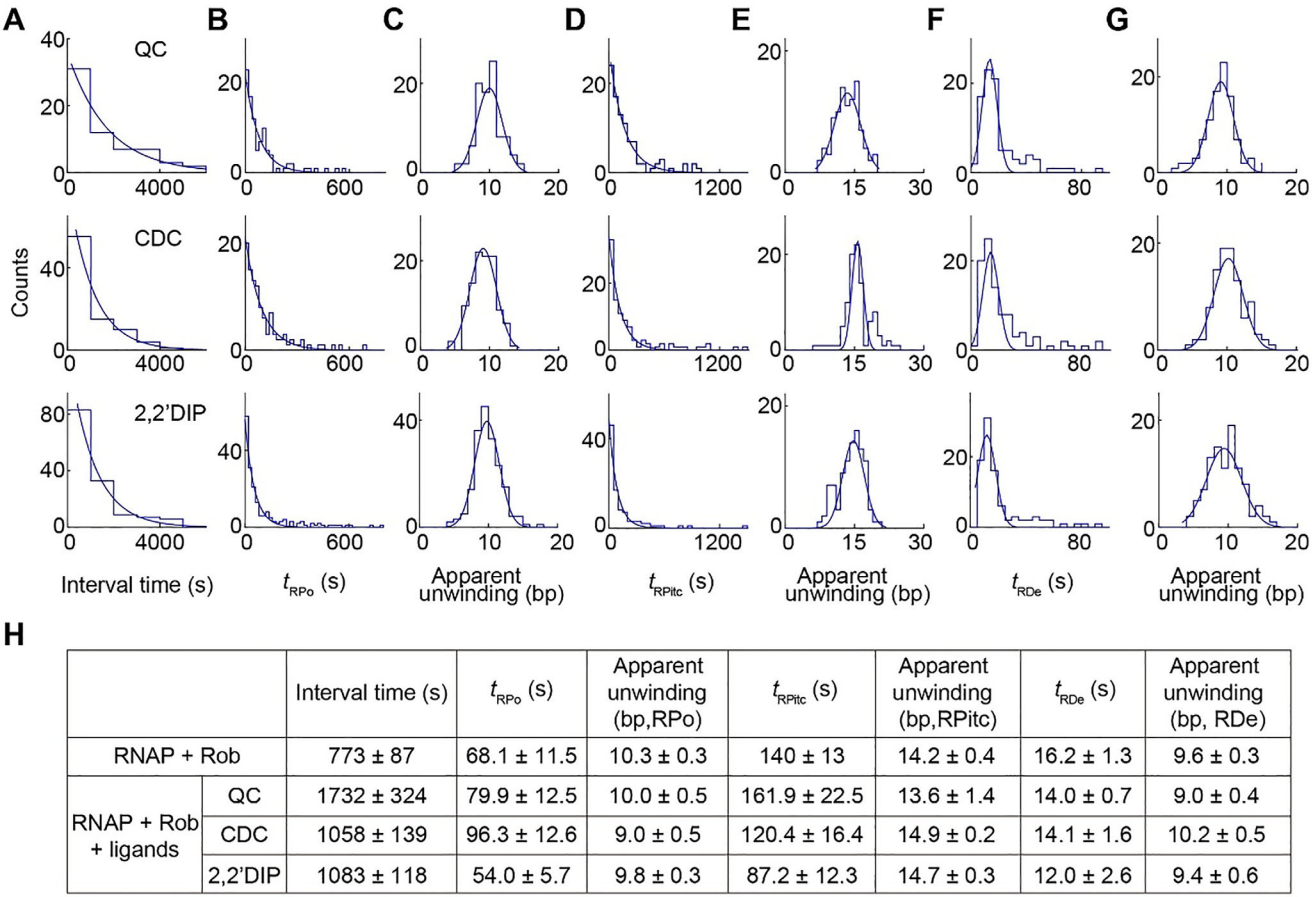
Characterization of ligand effect on Rob‐facilitated transcription. Three potential ligands (QC, CDC, and 2,2’DIP) were selected, and their effects on the kinetics of the states of RPo, RPitc, RDe, and their corresponding transcription bubble sizes were measured at 10 µm each of small ligands, 2 nm RN, AP, and 2 nm Rob on positively supercoiled DNA via single‐molecule magnetic trapping assays. Fitting each of the lifetime histograms to a single exponential function yields interval time of 1732 ± 324 s, 1058 ± 139 s, 1083 ± 118 s (SEM, *N* = 62, 86, 139 for (A), respectively; and *t*_RPo_ of 79.9 ± 12.5 s, 96.3 ± 12.6 s, 54.0 ± 5.7 s (SEM, *N* = 96, 109, 186 for (B), respectively; and the transcription bubble sizes of RPo states are fit to a single‐Gaussian function yielding peaks at 10.0 ± 0.5 bp, 9.0 ± 0.5 bp, 9.8 ± 0.3 bp (SEM, *N* = 96, 101, 101 for (C), respectively. (D) Fitting each of the RPitc lifetime histograms to a single exponential function yields *t*_RPitc_ of 161.9 ± 22.5 s, 120.4 ± 16.4 s, and 87.2 ± 12.3 s (SEM, *N* = 96, 101, 101), respectively. (E) Fitting the transcription bubble sizes to a single‐Gaussian function yields peaks at 13.5 ± 0.7 bp, 15.7 ± 1.3 bp, 14.7 ± 0.6 bp (SEM, *N* = 96, 101, 101), respectively. (F) The mean elongation time is calculated by fitting RDe time to a single‐Gaussian function, yielding peaks for *t*_RDe_ of 13.6 ± 1.4 s, 14.1 ± 1.6 s, and 12.0 ± 2.6 s (SEM, *N* = 96, 101, 101), respectively. (G) The corresponding transcription bubble sizes of RDe states are 9.0 ± 0.4 bp, 10.2 ± 0.5 bp, and 9.4 ± 0.6 bp (SEM, *N* = 96, 101, 101), respectively. (H) A table summarizing the fit parameters is attached.

We then employed single‐molecule magnetic trapping assays to characterize the kinetics of RNAP promoter search, RPo dissociation, and promoter escape under the effect of Rob bound to three ligands, quinoline carboxamide (QC), chenodeoxycholic acid (CDC), and 2,2′‐bipyridine (2‐2’DIP), respectively. These three ligands were selected because of their potential binding activity to Rob CTD [19], and their regulatory activity in the expression of bacterial genes mediated by AraC/XylS family regulators [36, 37]. Control experiments have been performed with 2 nm RNAP holoenzyme, 100 µm NTPs, and 10 µm each of the three ligands under positively supercoiled DNA, showing an insignificant effect of these three ligands on RNAP transcription in the absence of Rob (Figure S10). To verify their effect on Rob regulation, the lifetimes of different transcription states are analyzed and fit to a single exponential function, separately. The presence of quinoline carboxamide significantly enhances the mean interval time to 1732.4 ± 324 s (SEM), a value statistically identical to that of the promoter search by RNAP in the absence of Rob (Figure 5A and Table 3). This suggests that the Rob‐facilitated promoter search kinetics are possibly abolished by quinoline carboxamide. Chenodeoxycholic acid and 2,2′‐bipyridine slightly enhance the mean interval times to 1057.5 ± 139 s and 1082.6 ± 118 s (SEM), respectively, suggesting a relatively weak effect on the kinetics of Rob‐facilitated promoter search. Considering the previous reports that Rob CTD protects its NTD from binding to promoter DNA [19], our results may suggest that the binding of small ligands possibly causes conformational changes of Rob CTD to alter the affinity of Rob bound to promoter DNA. For the RPo state, we obtained statistically identical time constants in the presence of quinoline carboxamide or 2,2′‐bipyridine, respectively, but a significantly enhanced value in the presence of chenodeoxycholic acid, compared to that value measured in the presence of 2 nm RNAP and 2 nm Rob. This suggests a slightly stabilized RPo state by chenodeoxycholic acid, consistent with prior reports [37] (Figure 5B). The presence of either quinoline carboxamide or chenodeoxycholic acid doesn't significantly affect the time for the RPitc state (*t*_RPitc_, Figure 5D). However, the presence of 2,2′‐bipyridine significantly reduced *t*_RPitc_, suggesting accelerated kinetics of RNAP promoter escape. The transcription bubble size of the RPo state, or the RPitc state, or the RDe state, or the transcription elongation rate under positively supercoiled DNA is not affected by each of the three ligands (Figure 5C,E–G).

**TABLE 3:** Fit results on the effects of the three ligands on interval time, *t*_RPo,_ *t*_RPitc,_ *t*_RDe_, and transcription bubble sizes.

	QC	CDC	2,2’DIP
Interval time	1732 ± 324 s	1058 ± 139 s	1083 ± 118 s
*t*_RPo_	79.9 ± 12.5 s	96.3 ± 12.6 s	54.0 ± 5.7 s
Apparent unwinding	10.0 ± 0.5 bp	9.0 ± 0.5 bp	9.8 ± 0.3 bp
*t*_RPitc_	161.9 ± 22.5 s	120.4 ± 16.4 s	87.2 ± 12.3 s
Apparent unwinding	13.6 ± 1.4 bp	14.9 ± 0.2 bp	14.7 ± 0.3 bp
*t*_RDe_	14.0 ± 0.7 s	14.1 ± 1.6 s	12.0 ± 2.6 s
Apparent unwinding	9.0 ± 0.4 bp	10.2 ± 0.5 bp	9.4 ± 0.6 bp

## 3 Discussion

**FIGURE 6:**
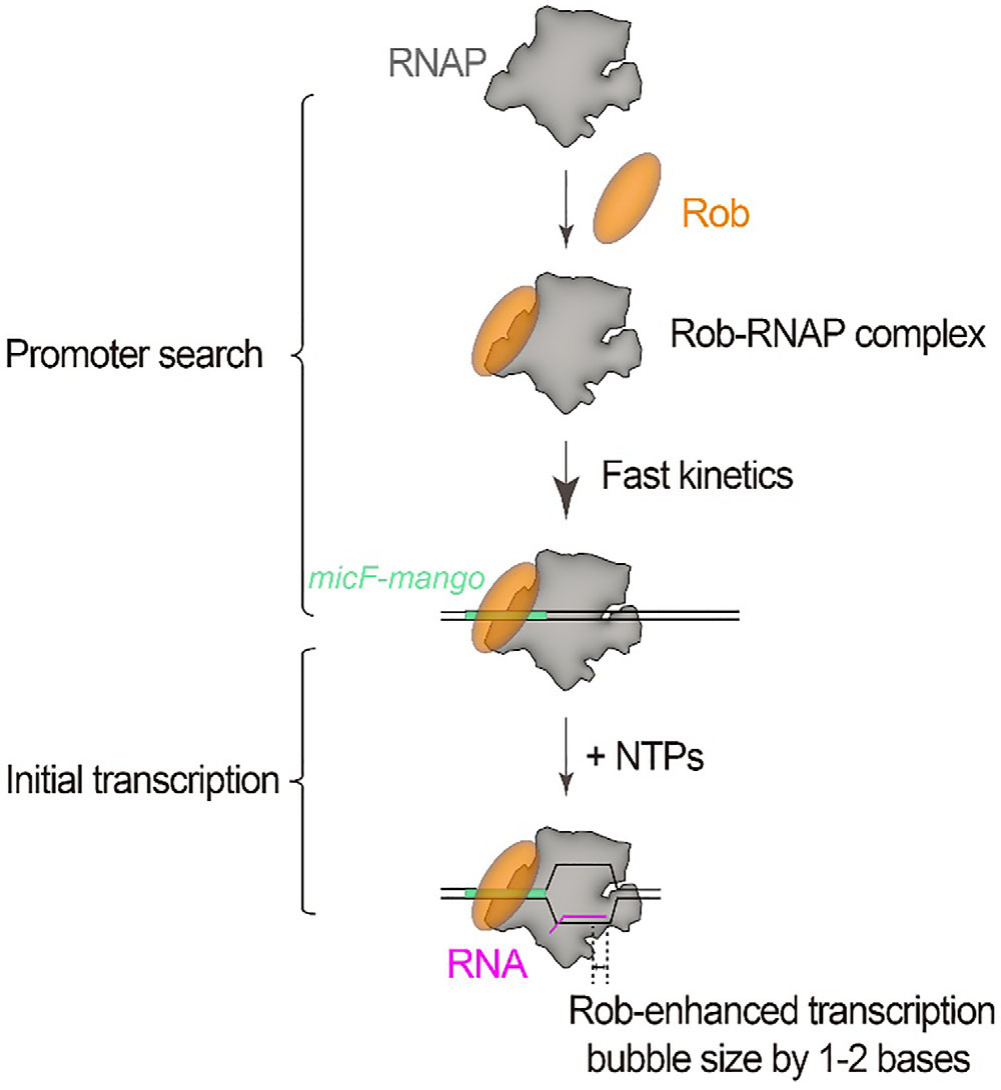
Model of Rob‐facilitated RNAP transcription derived from this study.

We report a factor‐dependent transcription activation mechanism for the pleiotropic AraC/XylS family regulator Rob. Taking the advantages of single‐molecule fluorescence assays and magnetic trapping assays in characterizing transcription kinetics, we find that Rob activates *E. coli* transcription by regulating the steps of promoter search and initial transcription (Figure 6), which will shed light on the regulation mechanisms of AraC/XylS family factors as well as the mechanisms of factor‐dependent transcription activation in prokaryotes and eukaryotes.

During the promoter search step, Rob prebinds to an RNAP molecule to form a Rob‐RNAP binary complex to accelerate the kinetics of promoter search. From the structural view of the Rob‐bound RPo complex as previously reported [19], Rob NTD can bind to the αCTD of RNAP, allowing the formation of the Rob‐RNAP binary complex. This Rob‐RNAP binary complex diffuses in solution to search promoters at a similar rate as a single RNAP molecule because of the insignificantly changed molecular size of Rob‐RNAP binary complex compared to RNAP alone [33, 38]. The Rob‐RNAP binary complex possibly possesses a higher binding affinity to the *micF‐mango* promoter than a single RNAP molecule due to the additional interactions between Rob and the Rob binding box, although the interaction between σ^70^R4 of RNAP and the −35 element of the promoter is excluded. Similar interactions with RNAP have been reported for other AraC/XylS regulators, MarA and SoxS [20, 39]. However, different from these AraC/XylS regulators, Rob has an additional CTD, which may bind small ligands with its pocket‐like architecture [19, 36, 37]. Our results show that the presence of quinoline carboxamide, chenodeoxycholic acid, or 2,2′‐bipyridine can significantly reduce the kinetics for promoter search, which not only supports their effects on the promoter search of Rob‐RNAP binary complex, but also supports the critical roles of Rob CTD in the facilitated promoter search of Rob‐RNAP binary complex.

After binding the promoter DNA, the Rob‐RNAP binary complex exhibits a slightly slower kinetic rate in forming the RPo state than a single RNAP does. This could possibly be because of the loss of the −35 element‐σ^70^R4 interaction during RPo formation by the Rob‐RNAP binary complex, whose interaction is, however, critical for *E. coli* RNAP RPo formation [7, 12, 19]. Even though the promoter‐bending property of Rob has been reported previously [19, 21, 22], this property unlikely favors the formation, stabilization or destabilization of the RPo state. In the presence of NTPs, Rob slightly promotes RNAP transition into RPitc state and significantly shortens the time that RNAP spends in the RPitc state before escaping from the promoter. During the RPitc state, Rob enhances the transcription bubble size by around 2 bases under negatively supercoiled DNA, allowing a more extended RNA transcript that favors initial transcription and promoter escape [34, 40, 41, 42]. However, the effects of Rob differ between negatively and positively supercoiled DNAs, which may partially reflect assay‐imposed topological constraints on Rob function. Further investigations would be required to elucidate the mechanism of Rob action in vivo. Our results show that small ligands can regulate the kinetics of Rob‐RNAP binary complex in the RPo state and the RPitc state, in agreement with the conclusion that small ligands can not only affect Rob‐dependent transcription activation but also raise the importance of Rob CTD in regulating the kinetics of RPo and RPitc states. Interestingly, we find that Rob slightly affects the kinetics of transcription elongation under negatively supercoiled DNA but not under positively supercoiled DNA, possibly suggesting a different mechanism of Rob regulation on negatively and positively supercoiled DNAs.

The activity of Rob in transcription activation is affected allosterically by the binding of small ligands to the pocket‐like architecture of Rob CTD, quinoline carboxamide, chenodeoxycholic acid, or 2,2′‐bipyridine, as we have tested. Interestingly, different ligands regulate transcription kinetics differently. Quinoline carboxamide tends to inhibit Rob‐dependent transcription activation, where it completely abolishes the Rob‐dependent acceleration of promoter search and weakly affects the kinetics of RPo and RPitc states. Chenodeoxycholic acid has a weak effect on Rob‐dependent transcription activation, where it slightly abolishes the Rob‐dependent promoter search and stabilizes the RPo state, which may facilitate entry into transcription initiation but insignificantly promotes promoter escape of RNAP [37]. 2,2′‐bipyridine slightly abolishes the Rob effect in promoter search but significantly enhances the kinetics RPitc states, consistent with previous reports that 2,2′‐bipyridine enhances Rob‐mediated transcription [36]. These results indicate diverse regulation mechanisms of Rob CTD under the effects of ligands, where a ligand binding can regulate the kinetics of Rob‐facilitated promoter search of RNAP or stabilization of the RPo state or the kinetics of promoter escape to modulate transcription separately, which may shed light on the regulation mechanisms of small ligands on Rob‐dependent transcription activation.

## 4 Methods

### 4.1 Protein Purification

Histidine‐tagged RNAP holoenzyme and SNAP‐tagged RNAP were expressed and purified as previously described [43]. Briefly, cells overexpressing histidine‐tagged RNAP were lysed in lysis buffer (20 mm Tris‐HCl pH 8, 500 mm NaCl, 5% (v/v) glycerol). After debris removal by centrifugation, the supernatant was subjected to Ni^2^^+^ affinity chromatography (HisTrap HP column, GE Healthcare), followed by heparin‐based ion‐exchange chromatography (HiTrap Heparin HP column, GE Healthcare). Fractions containing the RNAP core enzyme were pooled and incubated with a three‐fold molar excess of recombinant σ^70^. The mixture was dialyzed against storage buffer (20 mm Tris‐HCl pH 8, 200 mm NaCl, 0.1 mm EDTA, 1 mm DTT, 50% (v/v) glycerol), aliquoted, flash‐frozen in liquid nitrogen, and stored at −80°C.

Recombinant σ^70^ was prepared as follows [5]: σ^70^ was overexpressed in *E. coli* BL21(DE3) cells, lysed in Ni^2+^ buffer (20 mm Tris‐HCl pH 8, 500 mm NaCl, 5 mm imidazole, 5% (v/v) glycerol, 0.1 mm EDTA), and clarified by centrifugation. The supernatant was loaded onto a HisTrap HP Ni^2^^+^ affinity column (GE Healthcare), and bound proteins were eluted with a 15 mL linear imidazole gradient up to 250 mm. Eluted fractions were pooled, treated with PreScission Protease (PPX), and dialyzed overnight against Ni^2^^+^ buffer. The dialyzed sample was reapplied to a HisTrap HP column to remove uncleaved proteins and residual protease. The flow‐through containing σ^70^ was further purified by anion‐exchange chromatography (HiTrap Q column, GE Healthcare) and size‐exclusion chromatography (HiLoad Superdex 200 column, GE Healthcare). Purified σ^70^ was pooled, flash‐frozen in liquid nitrogen, and stored at −80°C. PPX was prepared as previously described [44].

Rob was prepared as previously described [19]. In brief, *E. coli* BL21(DE3) cells harboring the pET28a‐*rob* plasmid or its derivatives were cultured in 1 L of LB medium supplemented with 50 µg/mL kanamycin at 37°C. Cells were harvested by centrifugation at 5,422 × *g* for 10 min at 4°C, and the resulting pellet was resuspended in 20 mL of lysis buffer (20 mm Tris‐HCl pH 8.0, 200 mm NaCl, 5% glycerol). Cell was lysed and clarified by centrifugation at 13,000 × *g* for 40 min at 4°C. The supernatant was subjected to Ni‐NTA affinity chromatography using a 3 mL column (Qiagen, Inc.), followed by washing with 30 mL of buffer containing 40 mm imidazole and elution with 15 mL of buffer containing 200 mm imidazole. The eluted protein was further purified by size‐exclusion chromatography on a HiLoad 16/600 Superdex 200 column (GE Healthcare) equilibrated with buffer (20 mm Tris‐HCl pH 8, 75 mm NaCl, 5 mm MgCl_2_), using isocratic elution with 120 mL of the same buffer. Fractions containing Rob were pooled and stored at −80°C.

The plasmid of ybbR‐tagged Rob was prepared by inserting a ybbR‐tag followed by a 10x his tag at the C terminus of Rob. Purification was performed following the same procedure as described above.

SFP was prepared as previously reported [45].

### 4.2 Fluorescent Labeling of SNAP‐Tagged RNAP

SNAP‐tagged RNAP was fluorescently labeled as previously described [43]. Briefly, SNAP‐Surface 649 (New England Biolabs) was dissolved in DMSO at 1 mm concentration for stock. SNAP‐tagged RNAP and SNAP‐Surface 649 were mixed at a molar ratio of 1:2 in SNAP‐tagged RNAP storage buffer (20 mm Tris‐HCl pH 8, 200 mm NaCl, 0.1 mm EDTA, and 1 mm DTT, 10% (v/v) glycerol). The mixture was incubated at 4°C overnight in the dark and then separated via size‐exclusion chromatography (Biogel P6, Bio‐Rad). Proteins were aliquoted, flash frozen in liquid nitrogen, and stored at −80°C.

Rob was fluorescently labeled using Sfp phosphopantetheinyl transferase as described [45, 46, 47]. Briefly, Alexa546 maleimide or Alexa647 maleimide (Invitrogen) was dissolved in DMSO at 30 mm concentration for stock. CoA (Sigma) was dissolved in 1x PBS and incubated with the dye at an equal molar ratio at a final concentration of 15 mm at room temperature overnight. The reaction was stopped by adding 5 mm DTT, and the product was aliquoted and stored at ‐20°C. Rob‐ybbR was incubated with CoA‐Alexa546 or CoA‐Alexa647 at a molar ratio of 1:5 in buffer (20 mm Hepes K^+^, 150 mm NaCl, 10 mm MgCl_2_, 10% (v/v) glycerol, 5 µm Sfp, 1 mm TCEP) at 4°C overnight. Rob was further purified with a Mono S column (GE Healthcare) in a binding buffer (20 mm Hepes K^+^, 50 mm NaCl, 5 mm MgCl_2_, 1 mm DTT, and 10% (v/v) glycerol) and eluted in buffer (20 mm Hepes K^+^, 1 m NaCl, 5 mm MgCl_2_, 1 mm DTT, and 10% (v/v) glycerol). Peak fractions were collected, aliquoted, and stored at −80°C.

### 4.3 DNA Constructs for Single‐Molecule Magnetic Trapping Assays

DNA fragments flanked by *Kpn*I restriction sites were designed and synthesized (*micF‐mango*‐*his* in Table 4, Sangon Biotech). They contained a *micF*‐mango promoter (underlined), a 97‐bp transcription unit, and an *Escherichia coli his* terminator (bold), enabling RNAP to initiate transcription, elongate, and terminate. Following digestion with *Kpn*I restriction enzyme (New England Biolabs), the fragment was subcloned into a pre‐engineered pUC18 vector via *Kpn*I sites [44]. The resulting plasmid pUC18‐*micF*‐mango‐his was subsequently isolated from overnight *E. coli* cultures using the NucleoBond Xtra Midi Plus system (Macherey‐Nagel) through sequential alkaline lysis and anion‐exchange chromatography.

**TABLE 4:** DNA oligos used in this study.

Primer name	Sequence
*micF‐mango*‐*his*	5’‐GGTACCAAGCTAATAACTTATTCTTAAGTATTTGACAGCACTGAATGTCAAAACAAAACCTTCACTCGCAACTAGAATAACTCCCGCTATCATCATTAACTTTATCTAGTATGCATCGAATAGCCATCCCAATCGATATCGAGGAGTTTAAATATGGCTGATGCATGAATTCGTTAATAACGAAAGCCCCCGGAAGATGCATCTTCCGGGGGCTTTTTTTTTGGGAATTCGGTACC
biotin‐*micF‐mango* promoter‐F‐Cy3	5’‐AATAACTTATTCTTAAGTATTTGACAGCACTGAATGTCAAAACAAAACCTTCACTCGCAACTAGAATAACTCCCGCT(Cy3)ATCATCATTAACTTTATTATCGACCGTCGCCGTAAACTGC
*micF‐mango* promoter‐R	5’‐GCAGTTTACGGCGACGGTCGATAATAAAGTTAATGATGATAGCGGGAGTTATTCTAGTTGCGAGTGAAGGTTTTGTTTTGACATTCAGTGCTGTCAAATACTTAAGAATAAGTTATT
*micF‐mango* promoter‐R‐Cy5	5’‐GCAGTTTACGGCGACGGTCGAT(Cy5)AATAAAGTTAATGATGATAGCGGGAGTTATTCTAGTTGCGAGTGAAGGTTTTGTTTTGACATTCAGTGCTGTCAAATACTTAAGAATAAGTTATT

*micF‐mango*‐*his* DNA construct was prepared by digesting the pUC18‐*micF‐mango*‐*his* plasmid with XbaI and SbfI (New England Biolabs) and isolating via gel purification and extraction (PCR cleanup kit, Macherey‐Nagel). The 2.2 kb DNA was then ligated to 1 kb DNA fragments modified with multiple biotin groups through the XbaI site and to 1 kb DNA fragments modified with multiple digoxigenin groups through the SbfI site, respectively [25].

### 4.4 Single‐Molecule Magnetic Trapping Assays

Glass surfaces were subjected to standard cleaning procedures prior to assembly into flow chambers functionalized with anti‐digoxigenin antibodies (Roche) [25]. DNA molecules were tethered within these chambers between 1‐µm‐diameter superparamagnetic beads coated with streptavidin (Dynabeads MyOne Streptavidin C1; Life Technologies) and the glass surface. The assembled chamber was positioned on a custom‐built magnetic trap system that enabled real‐time manipulation and monitoring of bead positions to track changes in DNA extension. Raw data were collected at an acquisition frequency of 50 Hz and filtered at 0.5 Hz using the BeadTracker software suite developed by the N.H. Dekker laboratory [48]. Data analysis was performed using custom Matlab codes (http://github.com/Wang2004w/Code4DataAnalysis/tree/Seg2Analysis4MagneticTrappingAssayTranscription). Transcription experiments were conducted in transcription buffer containing 20 mm HEPES (Hydroxyethyl piperazine Ethane Sulfonic acid) K^+^ pH 8, 100 mm KGlut, 8 mm Mg(OAc)_2_, 0.5 mg/mL BSA, 0.1% (v/v) Tween 20, 10 µm ZnCl_2_, and 1 mm DTT at room temperature (approximately 28°C). DNA molecules maintained under controlled mechanical constraints (0.3 pN tension; 1 pN = 10^−^^1^ ^2^ N) exhibited defined superhelical densities, with corresponding positive supercoiling to σ = +0.023 (+5 turns).

### 4.5 DNA Constructs for Single‐Molecule Fluorescence Assays

DNA oligos containing *micF‐mango* promoter sequence (biotin‐*micF‐mango* promoter‐F‐Cy3, *micF‐mango* promoter‐R, and *micF‐mango* promoter‐R‐Cy5 in Table 2) were synthesized (Sangon Biotech) and dissolved in nuclease‐free water to 100 µM. Each pair of biotin‐*micF‐mango* promoter‐F‐Cy3/*micF‐mango* promoter‐R and biotin‐*micF‐mango* promoter‐F‐Cy3/*micF‐mango* promoter‐R‐Cy5 was annealed at 1:1 ratio and incubated at 95°C for 5 min, and then slowly cooled down to room temperature in about 2 h. These DNA constructs were aliquoted and stored at −20°C.

### 4.6 Single‐Molecule Fluorescence Assays

For single‐molecule fluorescence assays, a flow chamber was constructed using PEG‐modified surfaces as previously described [43] and subsequently positioned on a total internal reflection fluorescence (TIRF) microscope as previously described [49]. DNA molecules were immobilized onto these PEG‐modified surfaces through biotin‐streptavidin interactions. By washing out free components, an optimal density of fluorescent spots was obtained, feasible for single‐molecule detection and analysis. Experiments were conducted in a specialized transcription buffer consisting of 20 mm HEPES K^+^ pH 8, 100 mm KGlut, 8 mm Mg(OAc)_2_, 0.5 mg/mL BSA, 10 µµ ZnCl_2_, and 1 mm DTT. To minimize photobleaching and enhance fluorophore stability during extended imaging periods, this buffer was supplemented with an oxygen scavenging system comprising 1 mg/mL glucose oxidase, 0.4 mg/mL catalase, 0.8% (w/v) glucose, and 1 mm Trolox (Sigma‐Aldrich). Raw fluorescence intensity data from both donor (*I*_D_) and acceptor (*I*_A_) channels were extracted and subjected to background correction prior to analysis. FRET value was calculated as FRET = *I*_A_ / (*I*_D_ + *I*_A_).

### 4.7 Data Analysis for Single‐Molecule Magnetic Trapping Assays

Lifetimes for each transcription event were identified and manually analyzed using custom Matlab codes (http://github.com/Wang2004w/Code4DataAnalysis/tree/Seg2Analysis4MagneticTrappingAssayTranscription). The transcription bubble size of RPo, RPitc, and RDe states was analyzed by measuring the distance between each mean value of the RPo, RPitc, and RDe states and the DNA baseline. These values were converted to the number of base pairs unwound by RNAP by dividing each value by the slope of the linear regime of each DNA molecule when rotationally manipulating DNA molecules [30], and then multiplying by 10.4, the number of base pairs for one helical turn of DNA.

Lifetimes of the interval time between RPo states, RPo and RPitc states were each fit to a single exponential function,

(1) $$f\left(x\right)=A\times \exp\;\left(-\frac{x}{t_{0}}\right)$$

where *t*_0_ is the time constant for each state.

The mean time constants of the interval time between RPo states were fit to a Michaelis‐Menten model [50],

(2) $$f\left(x\right)=t_{0}\times \left(1+\frac{K_{m}}{x}\right)$$

where *t*_0_ is the time constant at saturate Rob concentration, *K*_m_ is the characteristic Rob concentration at which the time reaches half of *t*_0_.

The distribution of interval time between RPo states was globally fit to a single‐molecule Michaelis‐Menten model [50] to describe the two‐step process,

(3) $$f\left(x\right)=\frac{k_{1}k_{2}\cdot S}{k_{2}\;-\;k_{1}\cdot S}\times \left[\exp\left(-k_{1}\times S\times x\right)-\exp\left(-k_{2}x\right)\right]$$

where *k*_1_ and *k*_2_ were kinetic rate constants for Rob concentration‐dependent and independent processes, respectively. *S* is Rob's concentration, which is held during the fit.

Lifetimes of transcription elongation, the transcription bubble sizes of the RPo, RPitc, and RDe states were each fit to a single‐Gaussian function,

(4) $$f\left(x\right)=A\times \exp\left(-\;\frac{{\left(x-x_{0}\right)}^{2}}{width}\right)$$

where *x*_0_ is the time constant for elongation or the mean transcription bubble size for RPo, RPitc or RDe state.

### 4.8 Data Analysis for Single‐Molecule Fluorescence Assays

Lifetimes for each event of the appearance of RPo, RNAP, and their difference in Figure 2, and each event of the appearance of RPitc, RNAP, and their difference in Figure 3 were analyzed manually and fit to a single exponential function (Equation 1) to get their time constants. The FRET event of each condition was fit to a single‐Gaussian function (Equation 4) to obtain the mean FRET value (Figure 3C).

## Author Contributions

Y. Z., W. H., J. S., W. L., J. F., and S. W. designed research; Y. Z. and L. W. prepared reagents; Y. Z., W. H., J. S., L. W. performed research; Y. Z., C. T., and S. W. analyzed data; Y. Z., W. H., J. S., W. L., J. F., S. W. wrote the paper.

## Conflicts of Interest

The authors declare no conflict of interest.

## Supporting information




**Supporting File 1**: advs76334‐sup‐0001‐SuppMat.docx.

## Data Availability

The data that support the findings of this study are available from the corresponding author upon reasonable request.

## References

[1] D. F. Browning and S. J. W. Busby , “The Regulation of Bacterial Transcription Initiation,” Nature Reviews Microbiology 2 (2004): 57–65, 10.1038/nrmicro787. 15035009

[2] G. Zhang , E. A. Campbell , L. Minakhin , C. Richter , K. Severinov , and S. A. Darst , “Crystal Structure of Thermus Aquaticus Core RNA Polymerase at 3.3 Å Resolution,” Cell 98 (1999): 811–824, 10.1016/S0092-8674(00)81515-9. 10499798

[3] D. G. Vassylyev , S.‐I. Sekine , O. Laptenko , et al., “Crystal Structure of a Bacterial RNA Polymerase Holoenzyme at 2.6 Å Resolution,” Nature 417 (2002): 712–719, 10.1038/nature752. 12000971

[4] S. P. Haugen , W. Ross , and R. L. Gourse , “Advances in Bacterial Promoter Recognition and Its Control by Factors That Do Not Bind DNA,” Nature Reviews Microbiology 6 (2008): 507–519, 10.1038/nrmicro1912. 18521075 PMC3700611

[5] A. Feklistov and S. A. Darst , “Structural Basis for Promoter −10 Element Recognition by the Bacterial RNA Polymerase σ Subunit,” Cell 147 (2011): 1257–1269, 10.1016/j.cell.2011.10.041. 22136875 PMC3245737

[6] A. Chakraborty , D. Wang , Y. W. Ebright , et al., “Opening and Closing of the Bacterial RNA Polymerase Clamp,” Science 337 (2012): 591–595, 10.1126/science.1218716. 22859489 PMC3626110

[7] J. Chen , C. Chiu , S. Gopalkrishnan , et al., “Stepwise Promoter Melting by Bacterial RNA Polymerase,” Molecular Cell 78 (2020): 275–288.e6, 10.1016/j.molcel.2020.02.017. 32160514 PMC7166197

[8] S. C. Bera , P. P. B. America , S. Maatsola , et al., “Quantitative Parameters of Bacterial RNA Polymerase Open‐complex Formation, Stabilization and Disruption on a Consensus Promoter,” Nucleic Acids Research 50 (2022): 7511–7528, 10.1093/nar/gkac560. 35819191 PMC9303404

[9] R. M. Saecker , A. U. Mueller , B. Malone , et al., “Early Intermediates in Bacterial RNA Polymerase Promoter Melting Visualized by Time‐resolved Cryo‐electron Microscopy,” Nature Structural & Molecular Biology 31 (2024): 1778–1788, 10.1038/s41594-024-01349-9. PMC11821292 38951624

[10] A. Revyakin , C. Liu , R. H. Ebright , and T. R. Strick , “Abortive Initiation and Productive Initiation by RNA Polymerase Involve DNA Scrunching,” Science 314 (2006): 1139–1143, 10.1126/science.1131398. 17110577 PMC2754787

[11] A. N. Kapanidis , E. Margeat , S. O. Ho , E. Kortkhonjia , S. Weiss , and R. H. Ebright , “Initial Transcription by RNA Polymerase Proceeds through a DNA‐scrunching Mechanism,” Science 314 (2006): 1144–1147, 10.1126/science.1131399. 17110578 PMC2754788

[12] Y. Zhang , Y. Feng , S. Chatterjee , et al., “Structural Basis of Transcription Initiation,” Science 338 (2012): 1076–1080, 10.1126/science.1227786. 23086998 PMC3593053

[13] D. F. Browning and S. J. W. Busby , “Local and Global Regulation of Transcription Initiation in Bacteria,” Nature Reviews Microbiology 14 (2016): 638–650, 10.1038/nrmicro.2016.103. 27498839

[14] M. T. Gallegos , R. Schleif , A. Bairoch , K. Hofmann , and J. L. Ramos , “Arac/XylS family of Transcriptional Regulators,” Microbiology and Molecular Biology Reviews 61 (1997): 393–410. 9409145 10.1128/mmbr.61.4.393-410.1997 PMC232617

[15] D. Cortés‐Avalos , et al., “An Update of the Unceasingly Growing and Diverse AraC/XylS family of Transcriptional Activators,” Fems Microbiology Review 45 (2021): fuab020. 10.1093/femsre/fuab020 33837749

[16] H. J. Kwon , M. H. Bennik , B. Demple , and T. Ellenberger , “Crystal Structure of the Escherichia coli Rob Transcription Factor in Complex with DNA,” Nature Structural Biology 7 (2000): 424–430. 10802742 10.1038/75213

[17] K. L. Griffith , S. M. Becker , and E. Wolf , “Characterization of TetD as a Transcriptional Activator of a Subset of Genes of the Escherichia coli SoxS/MarA/Rob regulon,” Molecular Microbiology 56 (2005): 1103–1117, 10.1111/j.1365-2958.2005.04599.x. 15853893

[18] M. A. Zafar , I. M. Shah , and R. E. Wolf , “Protein–Protein Interactions between σ70 Region 4 of RNA Polymerase and Escherichia coli SoxS, a Transcription Activator That Functions by the Prerecruitment Mechanism: Evidence for “off‐DNA” and “on‐DNA” Interactions,” Journal of Molecular Biology 401 (2010): 13–32, 10.1016/j.jmb.2010.05.052. 20595001 PMC2917807

[19] J. Shi , F. Wang , F. Li , et al., “Structural Basis of Transcription Activation by Rob, a Pleiotropic AraC/XylS family Regulator,” Nucleic Acids Research 50 (2022): 5974–5987, 10.1093/nar/gkac433. 35641097 PMC9178005

[20] K. L. Griffith , I. M. Shah , T. E. Myers , M. C. O'Neill , and R. E. Wolf , “Evidence for “Pre‐Recruitment” as a New Mechanism of Transcription Activation in Escherichia coli: The Large Excess of SoxS Binding Sites per Cell Relative to the Number of SoxS Molecules per Cell,” Biochemical and Biophysical Research Communications 291 (2002): 979–986, 10.1006/bbrc.2002.6559. 11866462

[21] K. W. Jair , X. Yu , K. Skarstad , et al., “Transcriptional Activation of Promoters of the Superoxide and Multiple Antibiotic Resistance Regulons by Rob, a Binding Protein of the Escherichia coli Origin of Chromosomal Replication,” Journal of Bacteriology 178 (1996): 2507–2513, 10.1128/jb.178.9.2507-2513.1996. 8626315 PMC177972

[22] S. Rhee , R. G. Martin , J. L. Rosner , and D. R. Davies , “A Novel DNA‐Binding Motif in MarA: The First Structure for an AraC Family Transcriptional Activator,” Proceedings of the National Academy of Sciences 95 (1998): 10413–10418. 10.1073/pnas.95.18.10413 PMC27908 9724717

[23] A. Moreno , J. R. Froehlig , S. Bachas , et al., “Solution Binding and Structural Analyses Reveal Potential Multidrug Resistance Functions for SAV2435 and CTR107 and Other GyrI‐Like Proteins,” Biochemistry 55 (2016): 4850–4863, 10.1021/acs.biochem.6b00651. 27505298

[24] K. L. Griffith , M. M. Fitzpatrick , E. F. Keen , and R. E. Wolf , “Two Functions of the C‐Terminal Domain of Escherichia coli Rob: Mediating “Sequestration–Dispersal” as a Novel Off–On Switch for Regulating Rob's Activity as a Transcription Activator and Preventing Degradation of Rob by Lon Protease,” Journal of Molecular Biology 388 (2009): 415–430, 10.1016/j.jmb.2009.03.023. 19289129 PMC2728042

[25] A. Revyakin , R. H. Ebright , and T. R. Strick , “Single‐molecule DNA Nanomanipulation: Improved Resolution through Use of Shorter DNA Fragments,” Nature Methods 2 (2005): 127–138, 10.1038/nmeth0205-127. 16156080

[26] E. A. Galburt , S. W. Grill , A. Wiedmann , et al., “Backtracking Determines the Force Sensitivity of RNAP II in a Factor‐dependent Manner,” Nature 446 (2007): 820–823, 10.1038/nature05701. 17361130

[27] L. E. Tetone , L. J. Friedman , M. L. Osborne , et al., “Dynamics of GreB‐RNA Polymerase Interaction Allow a Proofreading Accessory Protein to Patrol for Transcription Complexes Needing Rescue,” Proceedings of the National Academy of Sciences 114 (2017): E1081–E1090, 10.1073/pnas.1616525114. PMC5320998 28137878

[28] H. R. Koh , R. Roy , M. Sorokina , et al., “Correlating Transcription Initiation and Conformational Changes by a Single‐subunit RNA Polymerase with near Base‐pair Resolution,” Molecular Cell 70 (2018): 695–706.e5, 10.1016/j.molcel.2018.04.018. 29775583 PMC5983381

[29] X. Chen , Q. Guo , J. Guan , et al., “Single‐molecule Tracking in Living Microbial Cells,” Biophysics Reports 11 (2025): 1, 10.52601/bpr.2024.240028. 40070662 PMC11891077

[30] A. Revyakin , R. H. Ebright , and T. R. Strick , “Promoter Unwinding and Promoter Clearance by RNA Polymerase: Detection by Single‐molecule DNA Nanomanipulation,” Proceedings of the National Academy of Sciences 101 (2004): 4776–4780, 10.1073/pnas.0307241101. PMC387324 15037753

[31] H. Hwang , H. Kim , and S. Myong , “Protein Induced Fluorescence Enhancement as a Single Molecule Assay with Short Distance Sensitivity,” Proceedings of the National Academy of Sciences 108 (2011): 7414–7418, 10.1073/pnas.1017672108. PMC3088603 21502529

[32] A. Mazumder , R. H. Ebright , and A. N. Kapanidis , “Transcription Initiation at a Consensus Bacterial Promoter Proceeds via a ‘Bind‐unwind‐load‐and‐lock’ mechanism,” eLife 10 (2021): e70090. 34633286 10.7554/eLife.70090 PMC8536254

[33] F. Wang , S. Redding , I. J. Finkelstein , J. Gorman , D. R. Reichman , and E. C. Greene , “The Promoter‐search Mechanism of Escherichia coli RNA Polymerase Is Dominated by Three‐dimensional Diffusion,” Nature Structural & Molecular Biology 20 (2012): 174–181, 10.1038/nsmb.2472. PMC3565103 23262491

[34] E. Lerner , S. Chung , B. L. Allen , et al., “Backtracked and Paused Transcription Initiation Intermediate of Escherichia coli RNA Polymerase,” Proceedings of the National Academy of Sciences 113 (2016): 6562–6571, 10.1073/pnas.1605038113. PMC5087071 27729537

[35] D. Duchi , D. L. V. Bauer , L. Fernandez , et al., “RNA Polymerase Pausing during Initial Transcription,” Molecular Cell 63 (2016): 939–950, 10.1016/j.molcel.2016.08.011. 27618490 PMC5031556

[36] J. L. Rosner , B. Dangi , A. M. Gronenborn , and R. G. Martin , “Posttranscriptional Activation of the Transcriptional Activator Rob by Dipyridyl in Escherichia coli,” Journal of Bacteriology 184 (2002): 1407–1416, 10.1128/JB.184.5.1407-1416.2002. 11844771 PMC134866

[37] E. Y. Rosenberg , D. Bertenthal , M. L. Nilles , K. P. Bertrand , and H. Nikaido , “Bile Salts and Fatty Acids Induce the Expression of Escherichia coli AcrAB Multidrug Efflux Pump through Their Interaction with Rob Regulatory Protein,” Molecular Microbiology 48 (2003): 1609–1619, 10.1046/j.1365-2958.2003.03531.x. 12791142

[38] L. J. Friedman , J. P. Mumm , and J. Gelles , “RNA Polymerase Approaches Its Promoter without Long‐range Sliding along DNA,” Proceedings of the National Academy of Sciences 110 (2013): 9740–9745, 10.1073/pnas.1300221110. PMC3683791 23720315

[39] R. G. Martin , W. K. Gillette , N. I. Martin , and J. L. Rosner , “Complex Formation between Activator and RNA Polymerase as the Basis for Transcriptional Activation by MarA and SoxS in *Escherichia coli* ,” Molecular Microbiology 43 (2002): 355–370, 10.1046/j.1365-2958.2002.02748.x. 11985714

[40] J. Brahms , O. Dargouge , S. Brahms , Y. Ohara , and V. Vagner , “Activation and Inhibition of Transcription by Supercoiling,” Journal of Molecular Biology 181 (1985): 455–465, 10.1016/0022-2836(85)90419-X. 2582135

[41] J. Ma , L. Bai , and M. D. Wang , “Transcription under Torsion,” Science 340 (2013): 1580–1583, 10.1126/science.1235441. 23812716 PMC5657242

[42] S. Kim , B. Beltran , I. Irnov , and C. Jacobs‐Wagner , “Long‐distance Cooperative and Antagonistic RNA Polymerase Dynamics via DNA Supercoiling,” Cell 179 (2019): 106–119.e16, 10.1016/j.cell.2019.08.033. 31539491

[43] E. T. Graves , C. Duboc , J. Fan , F. Stransky , M. Leroux‐Coyau , and T. R. Strick , “A Dynamic DNA‐repair Complex Observed by Correlative Single‐molecule Nanomanipulation and Fluorescence,” Nature Structural & Molecular Biology 22 (2015): 452–457, 10.1038/nsmb.3019. 25961799

[44] Y. Zhang , W. Han , L. Wang , et al., “Correlative *Escherichia coli* Transcription Rate and Bubble Conformation Remodeled by NusA and NusG,” The Journal of Physical Chemistry B 127 (2023): 2909–2917, 10.1021/acs.jpcb.2c08771. 36977198

[45] J. Yin , A. J. Lin , D. E. Golan , and C. T. Walsh , “Site‐specific Protein Labeling by SFP Phosphopantetheinyl Transferase,” Nature Protocols 1 (2006): 280–285, 10.1038/nprot.2006.43. 17406245

[46] B. R. Duewell , N. E. Wilson , G. M. Bailey , S. E. Peabody , and S. D. Hansen , “Molecular Dissection of PI3Kβ Synergistic Activation by Receptor Tyrosine Kinases, GβGγ, and Rho‐family GTPases,” eLife 12 (2024): RP88991. 38713746 10.7554/eLife.88991 PMC11076043

[47] M. K. Rathinaswamy , M. L. Jenkins , B. R. Duewell , et al., “Molecular Basis for Differential Activation of p101 and p84 Complexes of PI3Kγ by Ras and GPCRs,” Cell Reports 42 (2023): 112172, 10.1016/j.celrep.2023.112172. 36842083 PMC10068899

[48] J. P. Cnossen , D. Dulin , and N. H. Dekker , “An Optimized Software Framework for Real‐time, High‐throughput Tracking of Spherical Beads,” Review of Scientific Instruments 85 (2014): 103712, 10.1063/1.4898178. 25362408

[49] Y. Xiong , W. Han , C. Xu , et al., “Single‐molecule Reconstruction of Eukaryotic Factor‐dependent Transcription Termination,” Nature Communications 15 (2024): 5113, 10.1038/s41467-024-49527-z. PMC11180205 38879529

[50] S. C. Kou , B. J. Cherayil , W. Min , B. P. English , and X. S. Xie , “Single‐Molecule Michaelis−Menten Equations,” The Journal of Physical Chemistry B 109 (2005): 19068–19081, 10.1021/jp051490q. 16853459

