# Supplementary material for: Single‐Molecule Characterization of Bacterial Factor‐Dependent Transcription Activation by Rob
Source: Adv Sci (Weinh). 2026 Jul 6:e76334. Online ahead of print. doi: 10.1002/advs.76334 (PMC13335620; doi:10.1002/advs.76334)
Supplement: Supplementary file 1 — Supporting File 1: advs76334‐sup‐0001‐SuppMat.docx. [file ADVS-9999-e76334-s001.docx]

**SUPPLEMENTARY INFORMATION**

**Single-molecule characterization of bacterial factor-dependent transcription activation by Rob**

**Zhang et al.**


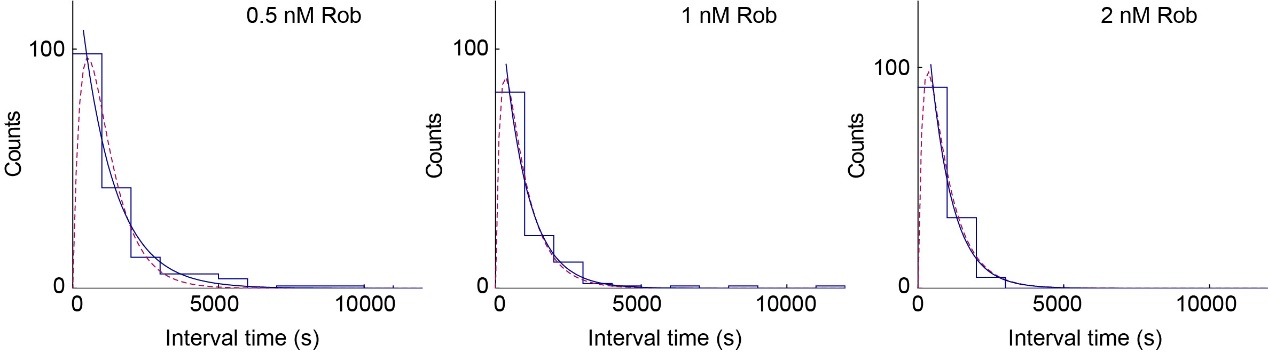


Supplementary Figure 1. Histograms of the interval time between RPo states achieved via single-molecule magnetic trapping assay were fit to the single-molecule Michaelis-Menten model, yielding *k*_1_ = 5.5 ± 1.0 M^-12^s^-1^ with *k*_2_ constraint as the inverse of *t*_0_ obtained from Figure 1F.


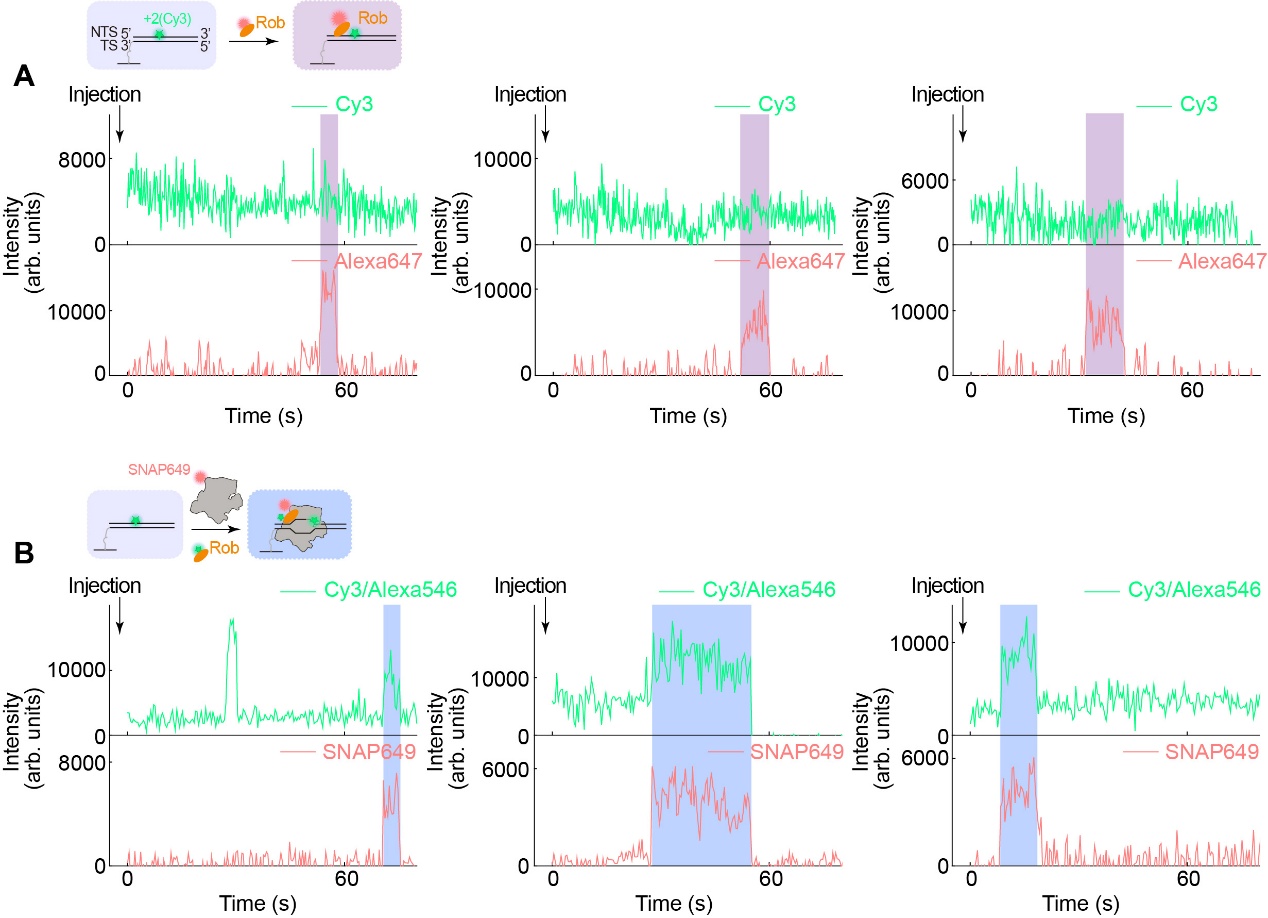


Supplementary Figure 2. Typical trajectories reflecting the binding of Rob-Alexa647 to DNA-Cy3 (A). Typical trajectories representing the simultaneous binding of Rob-Alexa546 and RNAP-SNAP649 to DNA-Cy3 (B).


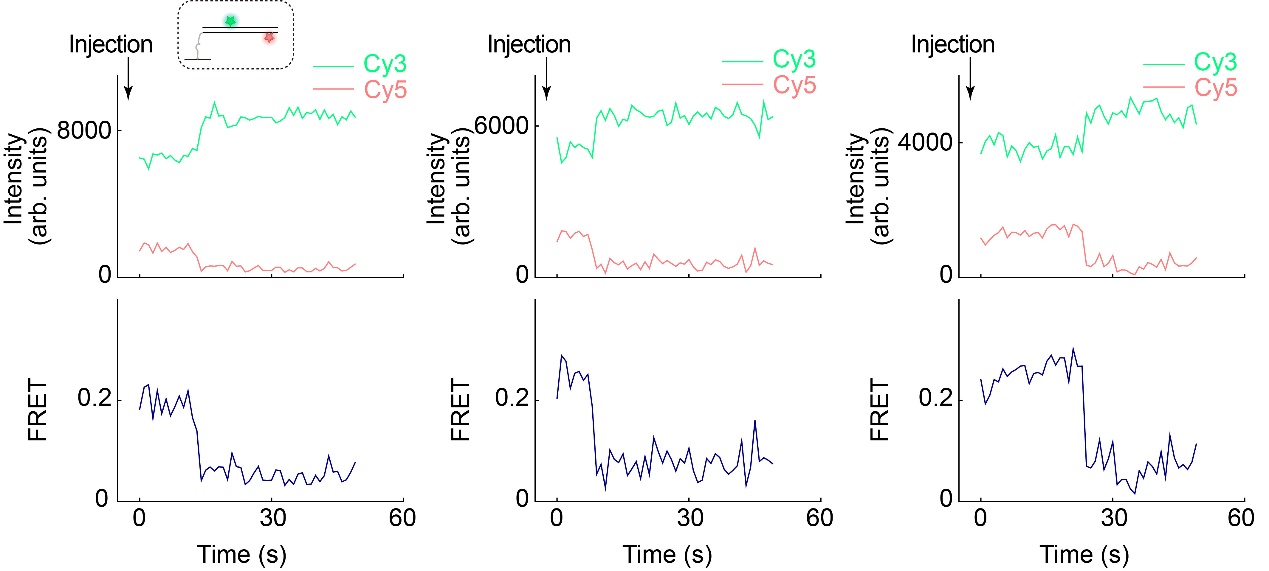


Supplementary Figure 3. Typical trajectories reflecting FRET values for surface-tethered DNA-Cy3/Cy5 as used in Figure 3.


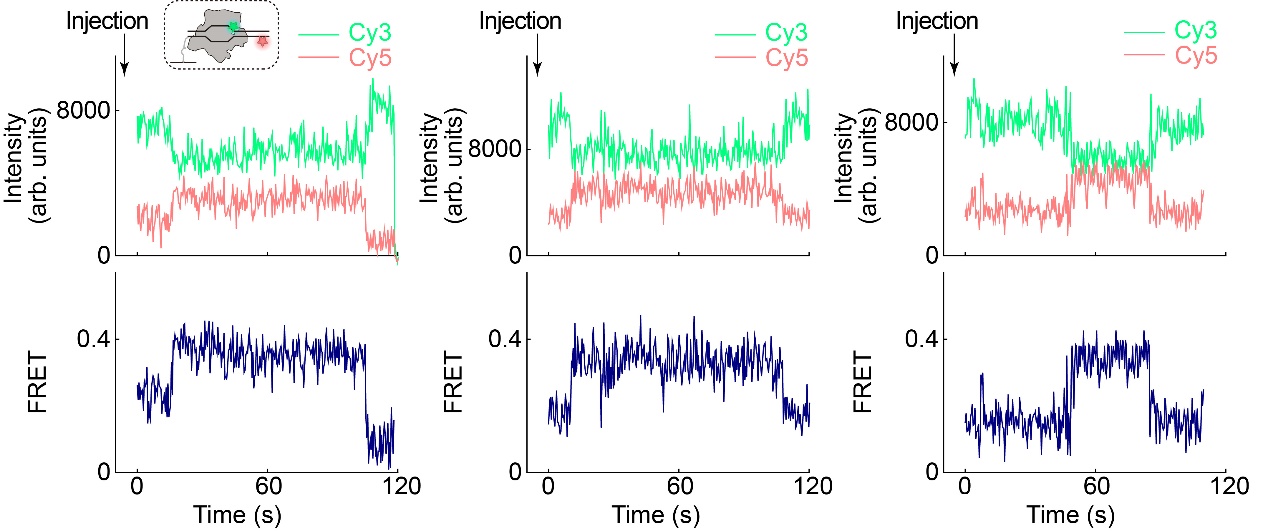


Supplementary Figure 4. Typical trajectories reflecting FRET changes for RPo formation on DNA-Cy3/Cy5.


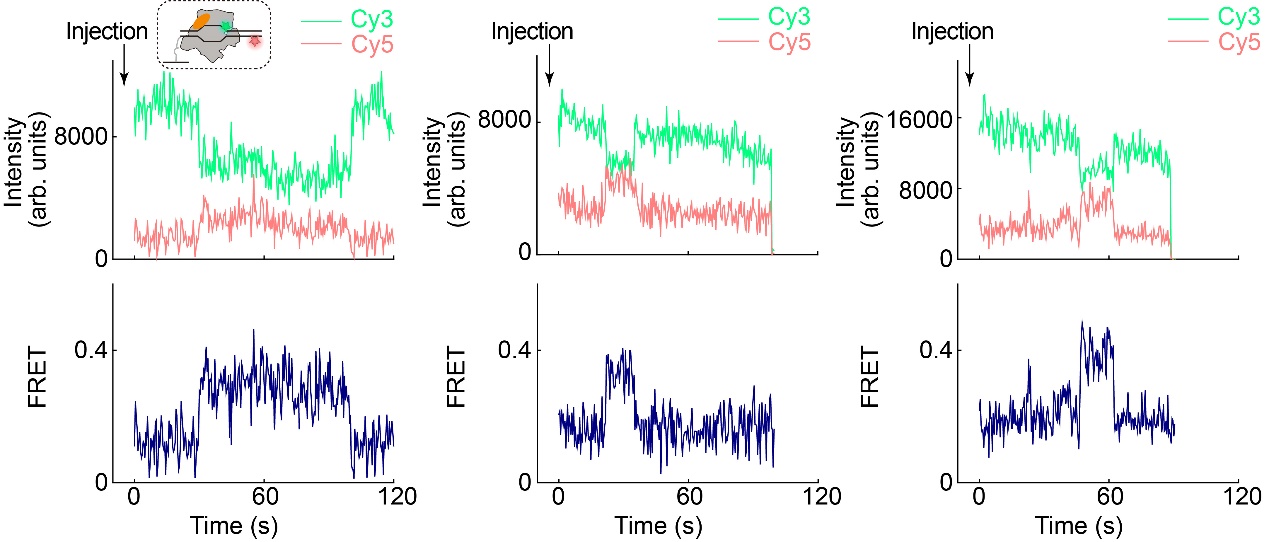


Supplementary Figure 5. Typical trajectories representing FRET changes upon RPo formation on DNA-Cy3/Cy5 in the presence of Rob.


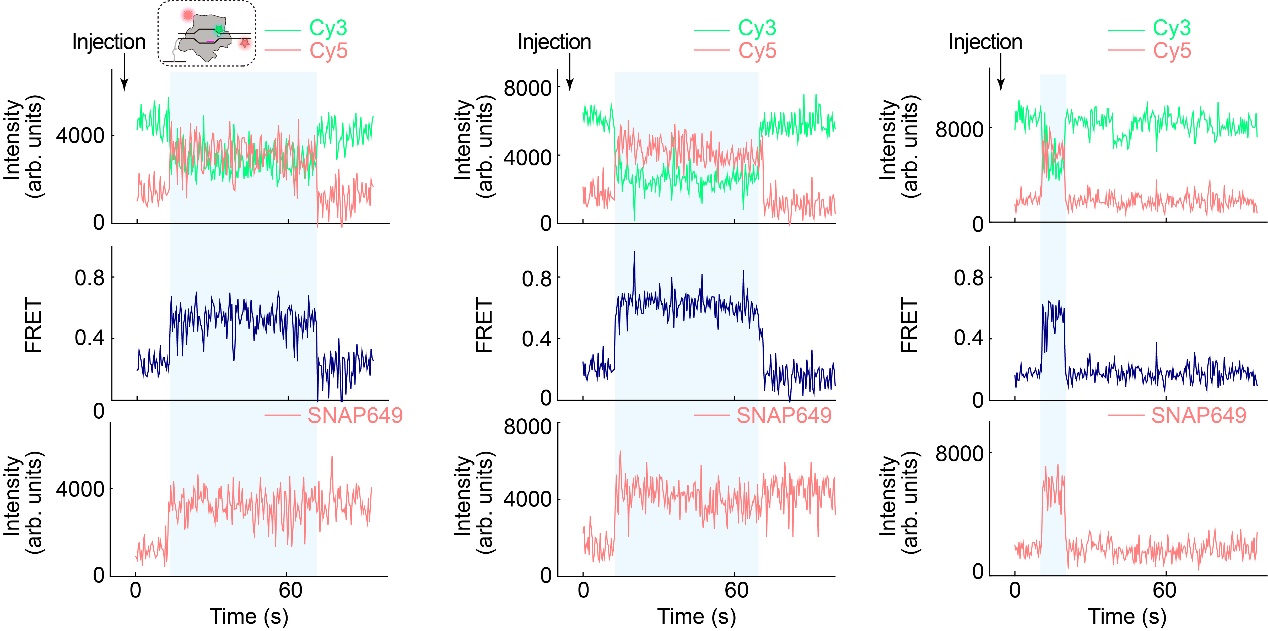


Supplementary Figure 6. Typical trajectories reflecting FRET changes for the RPitc formation on DNA-Cy3/Cy5 with RNAP-SNAP649.


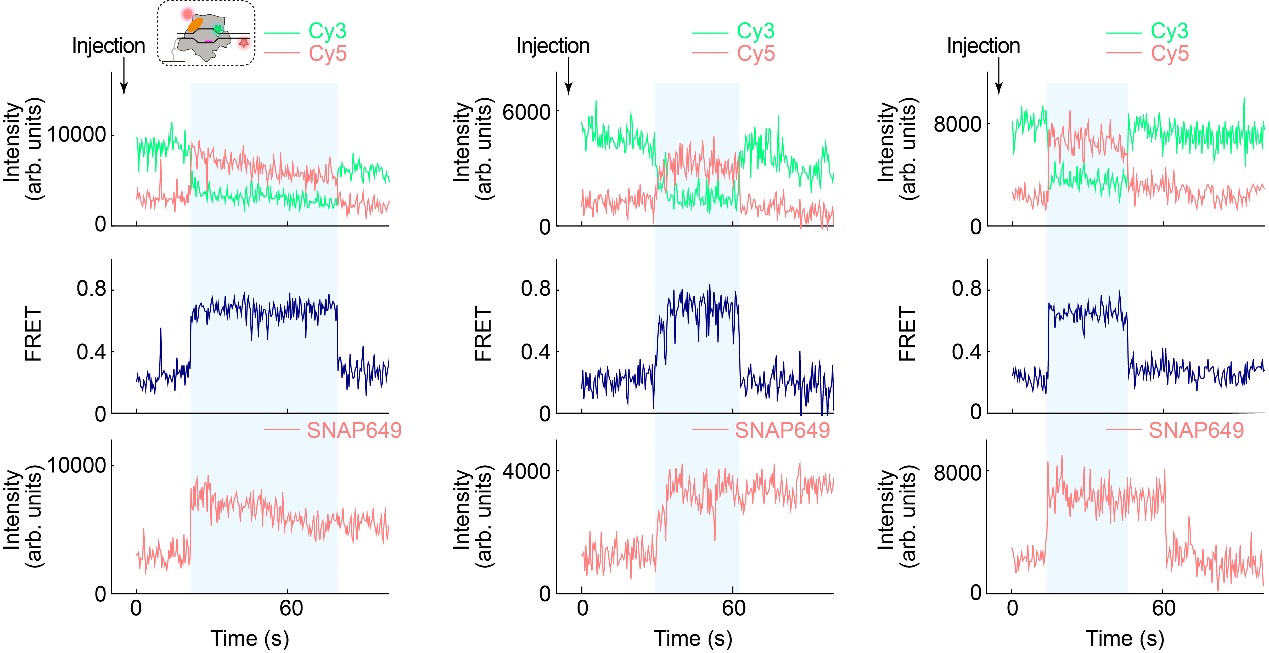


Supplementary Figure 7. Typical trajectories reflecting FRET changes for the RPitc formation on DNA-Cy3/Cy5 with RNAP-SNAP649 and Rob.


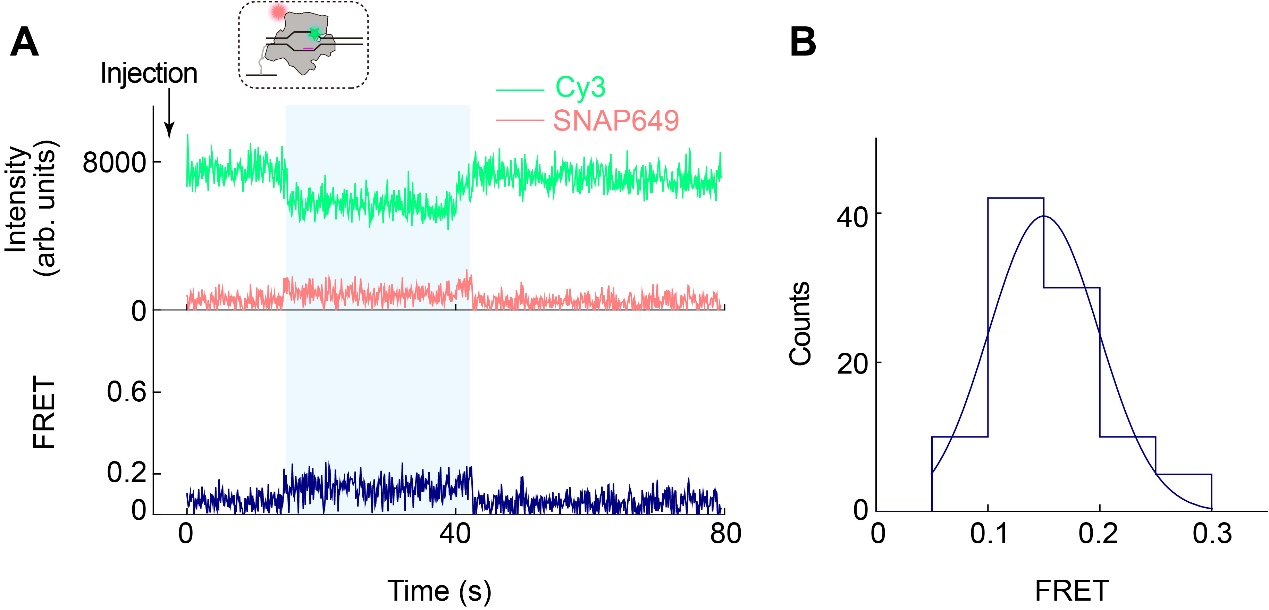


Supplementary Figure 8. (A) Typical trajectories reflecting FRET changes of RPitc formation on DNA-Cy3 with RNAP-SNAP649. (B) FRET histogram of the mean FRET value of the RPitc state of each trajectory was fit to a single Gaussian function yielding a peak at 0.15 ± 0.01 (SEM, *N* = 99).


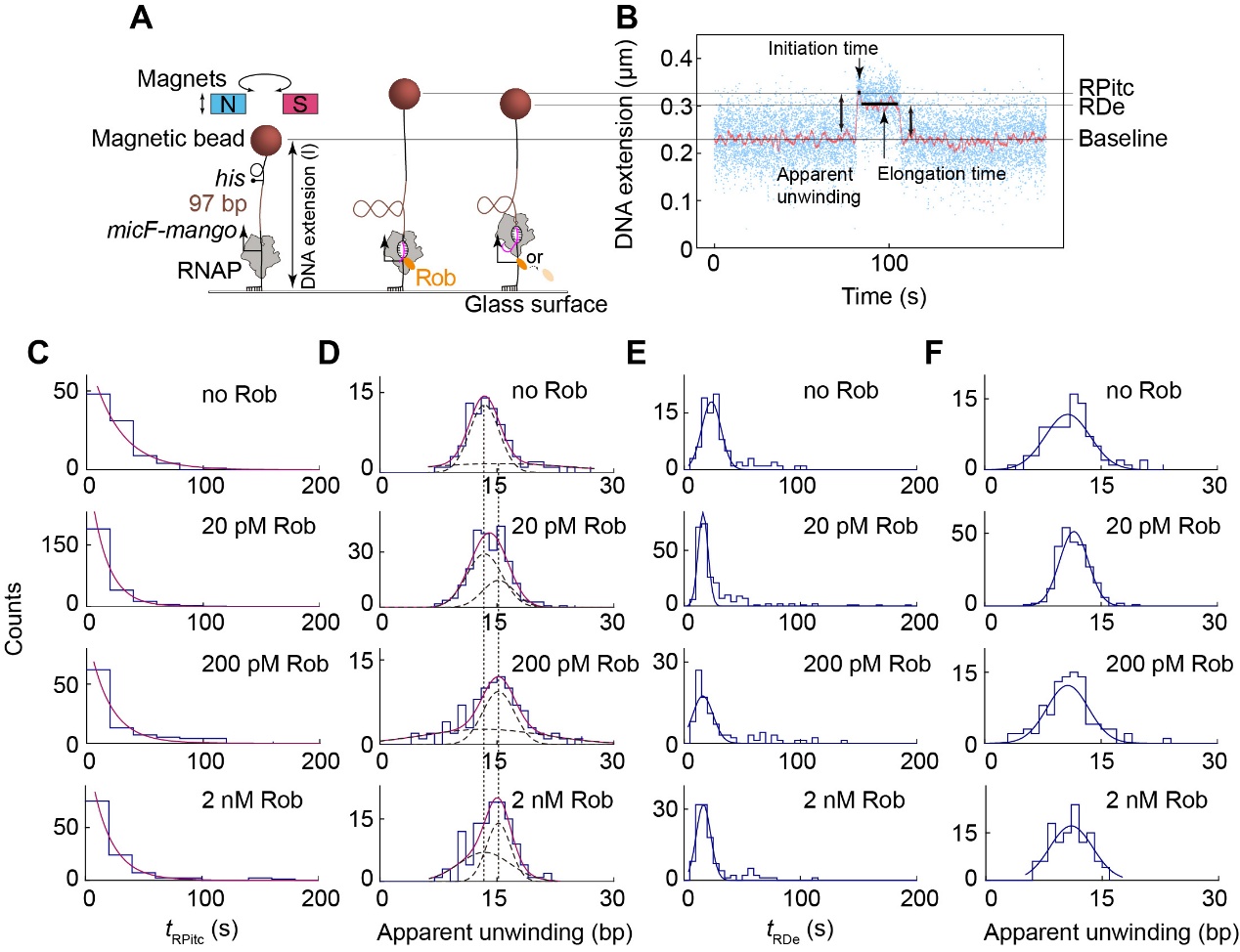


Supplementary Figure 9. Characterization of Rob effect on transcription initiation and elongation under negatively supercoiled DNA via single-molecule magnetic trapping assays.

(A) Schematic for the assay. (B) A typical trajectory representing the sequential formation of RPitc and RDe states at 20 pM RNAP concentration. (C) Histograms of the lifetime of each RPitc state are fit to a single exponential function giving time constants of 25.2 ± 3.2 s, 14.7 ± 1.1 s, 18.6 ± 2.7 s and 17.4 ± 2.0 s (SEM, *N* = 94, 252, 96, 118), respectively. (D) Histograms of the transcription bubble sizes are distributed and globally fit to a double-Gaussian function, yielding two peaks at 13.4 ± 0.3 bp and 15.2 ± 0.3 bp, respectively. The amplitudes of these two peaks are 12.7 ± 2.3 and 1.7 ± 1.1 for 0 nM Rob, 29.2 ± 6.7 and 14.7 ± 7.6 for 20 pM Rob, 2.7 ± 1.5 and 9.4 ± 2.3 for 200 pM Rob and 7.0 ± 3.2 and 14.0 ± 3.7 for 2 nM Rob. (E) Histograms of the lifetime of each RDe state are fit to a single-Gaussian function giving peaks at 23.4 ± 2.1 s, 16.3 ± 0.7 s, 16.1 ± 1.4 s and 16.8 ± 1.5 s (SEM, *N* = 94, 252, 96, 118), respectively. (F) Histograms of the transcription bubble sizes are fit to a single-Gaussian function yielding peaks at 10.7 ± 0.7 bp, 11.5 ± 0.3 bp, 10.7 ± 0.3 bp and 10.9 ± 0.7 bp (SEM, *N* = 94, 252, 96, 118), respectively. Data for 0 and 2 nM Rob are represented in Figure 4G- J.


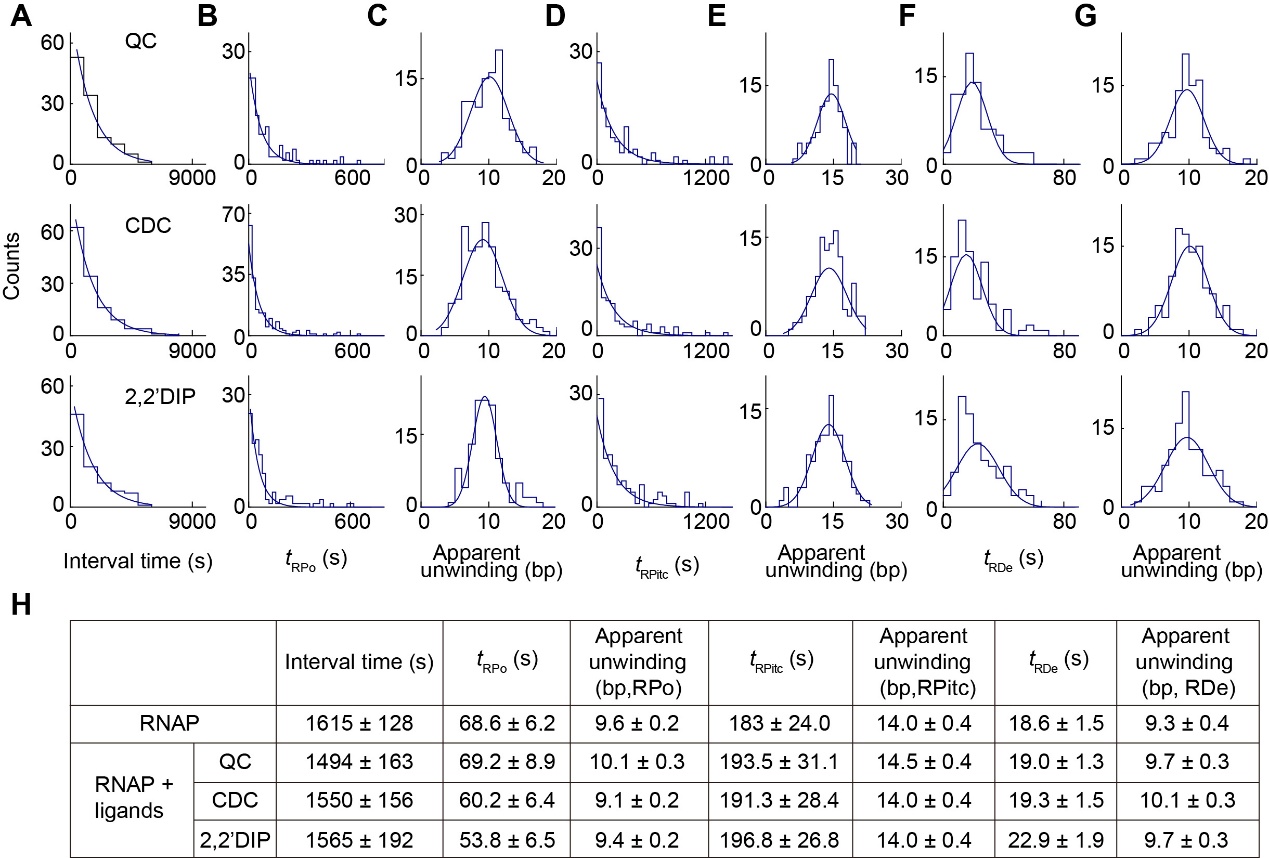


Supplementary Figure 10. Control experiments representing the effect of the ligands (QC, CDC and 2,2’DIP) on RNAP transcription via single-molecule magnetic trapping assays.

The experiment is performed in the absence of Rob but with each of the three ligands. Fitting each of the lifetime histograms to a single exponential function yields interval time of 1494 ± 163 s, 1550 ± 156 s, 1565 ± 192 s (SEM, *N* = 116, 131, 95 for A), respectively; and *t*_RPo_ of 69.2 ± 8.9 s, 60.2 ± 6.4 s, 53.8 ± 6.5 s (SEM, *N* = 113, 190, 118 for B), respectively; and the transcription bubble sizes of RPo states are fit to a single-Gaussian function yielding peaks at 10.1 ± 0.3 bp, 9.1 ± 0.2 bp, 9.4 ± 0.2 bp (SEM, *N* = 113, 190, 118 for C), respectively. (D) Fitting each of the RPitc lifetime histograms to a single exponential function yields *t*_RPitc_ of 193.5 ± 31.1 s, 191.3 ± 28.4 s, and 196.8 ± 26.8 s (SEM, *N* = 102, 112, 117), respectively. (E) Fitting the transcription bubble sizes to a single-Gaussian function yields peaks at 14.5 ± 0.4 bp, 14.0 ± 0.4 bp, 14.0 ± 0.4 bp (SEM, *N* = 102, 112, 117), respectively. (F) The mean elongation time is calculated by fitting RDe time to a single-Gaussian function yielding peaks for *t*_RDe_ of 19.0 ± 1.3 s, 19.3 ± 1.5 s, and 22.9 ± 1.9 s (SEM, *N* = 102, 112, 117), respectively. (G) The corresponding transcription bubble sizes of RDe states are 9.7 ± 0.3 bp, 10.1 ± 0.3 bp, and 9.7 ± 0.3 bp (SEM, *N* = 102, 112, 117), respectively. (H) A table summarizing the fitting parameters is attached.
